# Skeletal Morphology of *Opius dissitus* and *Biosteres carbonarius* (Hymenoptera: Braconidae), with a Discussion of Terminology

**DOI:** 10.1371/journal.pone.0032573

**Published:** 2012-04-30

**Authors:** Dave Karlsson, Fredrik Ronquist

**Affiliations:** Department of Entomology, Swedish Museum of Natural History, Stockholm, Sweden; United States Department of Agriculture, Agriculture Research Service, United States of America

## Abstract

The Braconidae, a family of parasitic wasps, constitute a major taxonomic challenge with an estimated diversity of 40,000 to 120,000 species worldwide, only 18,000 of which have been described to date. The skeletal morphology of braconids is still not adequately understood and the terminology is partly idiosyncratic, despite the fact that anatomical features form the basis for most taxonomic work on the group. To help address this problem, we describe the external skeletal morphology of *Opius dissitus* Muesebeck 1963 and *Biosteres carbonarius* Nees 1834, two diverse representatives of one of the least known and most diverse braconid subfamilies, the Opiinae. We review the terminology used to describe skeletal features in the Ichneumonoidea in general and the Opiinae in particular, and identify a list of recommend terms, which are linked to the online Hymenoptera Anatomy Ontology. The morphology of the studied species is illustrated with SEM-micrographs, photos and line drawings. Based on the examined species, we discuss intraspecific and interspecific morphological variation in the Opiinae and point out character complexes that merit further study.

## Introduction

The parasitic-wasp family Braconidae forms one of the most impressive insect radiations we know. Almost 18,000 species have been described to date [1] but recent estimates suggest that the true diversity may be in the range of 40,000 to 120,000 species [2], [3]. Thus, braconids constitute a tremendous challenge in current efforts to complete the biological inventory of the planet.

Despite the fact that most taxonomic work on braconids is based on external morphology, and will remain so for the foreseeable future, there is a lack of detailed morphological studies of these wasps. Ghahari & Achterberg [3] and Shenefelt [4] list almost 19,000 scientific papers discussing the Braconidae in their bibliographies but few of these papers cover the external morphology in any detail. One of the few exceptions is the description of the external and internal anatomy of *Stenobracon deesae* [5], [6], unfortunately published in an Indian journal that is not widely available. General taxonomic treatments of braconids [2], [7], [8], [9], [10], [11], [12], ichneumonids [13], [14], [15], [16], [17] or hymenopterans [18], [19], [20], [21] provide some information relevant to braconid morphology and terminology but lack the type of details found in the in-depth studies of exemplar species available for some other groups of hymenopterans (e.g., Snodgrass [22] (bees), Duncan [23] (vespids), Michener [24] (bees), Ronquist & Nordlander [25] (ibaliids)).

As a result, many of the characters used in taxonomic work on braconids are poorly understood or misinterpreted. For instance, Shenefelt [4] complained that most original descriptions of braconid species are poorly illustrated and many of them vaguely worded as well. It is true that the standard of braconid species descriptions have improved considerably over time: Linnaeus [26] used just three words to describe the braconid *Microgaster globata* (L.) (black, red feet) but modern descriptions often provide multifaceted descriptions backed by rich sets of relevant illustrations e.g. [27], [28], [29]. Nevertheless, the terminology for the different structures is not always consistent with that used for other insects, other hymenopterans or even other braconids, and a number of important character complexes remain underutilized as sources of informative characters in taxonomic and systematic papers on the group.

Although terminological confusion has reigned in the Hymenoptera in the past, detailed morphological studies of a number of character systems across a broad sample of taxa, such as those of Gibson [30], [31], [32], Vilhelmsen [33], Krogmann and Vilhelmsen [34] and Mikó et al. [35] on mesothoracic structures, Basibuyuk and Quicke [36] on the antennal cleaner, Oeser [37] and Vilhelmsen [38] on the ovipositor complex, and Schulmeister [39], [40] on the male genitalia, have contributed greatly to a more consistent terminology in the last decades. Unfortunately, there is a lack of easily accessible compilations of this information and of papers discussing how the general terminology ought to be applied in different groups. This is true for the Ichneumonoidea as well as for many other hymenopteran groups.

The monophyly of the Braconidae and its sister-group relationship with the Ichneumonidae are well established today [41]. In a seminal paper, van Achterberg [42] divided the Braconidae into four larger subdivisions, but this hypothesis is still highly controversial [3]. More than 40 subfamilies of braconids are currently recognized, several of them discovered or described within the last 15 years (see e.g. [3]). The relationships among the subfamilies have been the subject of considerable discussion [43], [44], [45] but remain difficult to resolve despite a number of recent molecular analyses [46], [47], [48].

Opiinae is one of the larger braconid subfamilies with more than 1,500 described species. The biology is known for about one third of the species, all of which are koinobiont endoparasitoids of cyclorrhaphous Diptera. Many species are of economic importance as biological control agents (e.g. [49], [50], [51]). They oviposit into the host egg or larva and emerge as adult wasps from the host puparium.

Fischer contributed greatly to the knowledge of the World fauna of opiines in a series of papers published between 1956 and 1983. A synthesis of a major portion of his work, including a brief overview of opiine morphology and terminology, appeared in a volume of Das Tierreich [52]. Other significant contributions include Eady [53], Tobias and Jakimavicius [54], Buckingham and Sharkey [55], Sharkey and Rasnitsyn [56], Wharton [57], [58], [59], and van Achterberg [e.g. [8], [9], [42], [60].

The monophyly of the clade consisting of the Opiinae and Alysiinae, the two braconid subfamilies that are exclusively endoparasitic on cyclorrhaphous Diptera, is firmly established both by morphological studies [42], [43], [44] and molecular analyses, e.g. [48], [61]. However, while the Alysiinae are characterized as a monophyletic group by their exodont mandibles and complete loss of the occipital carina, the Opiinae lack clear morphological synapomorphies (e.g. [2]) and may be paraphyletic with respect to the Alysiinae.

In this work, we describe the morphology of *Opius dissitus* Muesebeck, 1963 and *Biosteres carbonarius* Nees, 1834, two phylogenetically distant representatives of the Opiinae. The species *O. dissitus* was chosen both because we had access to an abundant supply of specimens, and because it is very similar morphologically to *Opius pallipes* Wesmael, 1835, the type species of *Opius*. The choice of *B. carbonarius* was based on the fact that its morphology is quite different from that of *O. dissitus*. In fact, morphological data indicate that it forms part of a substantial cluster of species in a lineage separate from other opiines, the Biosterina or Biosterini [9], [59], a hypothesis also supported by more recent molecular studies [61], [62], [63]. Based on the morphology of *O. dissitus* and *B. carbonarius*, and on comparisons with the morphology of other Hymenoptera reported in the literature, we attempt to define a reasonable terminology of external morphological structures for use in taxonomic work on opiines and other braconids. We also discuss the inter- and intraspecific variation of the studied characters in the subfamily Opiinae.

## Materials and Methods

The present study is based on dissection of 30 females and 48 males of *Opius dissitus* Muesebeck 1963 and 9 females and 3 males of *Biosteres carbonarius* Nees 1834. In addition, a large number of dry-mounted specimens of these and other Opiinae species were examined without dissection. All the *O. dissitus* specimens were reared by Amy Bader in Robert A. Wharton’s lab in the Department of Entomology, Texas A&M University, USA. The culture was originally obtained from Fred Petitt at Disney World’s Epcot Center in Florida and was reared upon an unspecified species of *Liriomyza* leaf miners (Diptera: Agromyzidae). The 12 specimens of *Biosteres carbonarius* Nees, were collected by the Swedish Malaise Trap Project (SMTP) as follows: 2 females from Sweden, Uppland, Knivsta kommun, Rickebasta alsumpskog, deciduous forest (N 59°44.061′ E 17°43.225′ trap ID 9, coll. ID 1608, 2005.v.28-2005.vi.11); 2 females from Sweden, Småland, Älmhults kommun, Stenbrohult, deciduous forest (N 56°36.548′ E 14°11.583′ trap ID 24, coll. ID 1310, 2004.vii.22-2004.ix.25); 1 female from Sweden, Halland, Stenungsunds kommun, Kolhättan, deciduous forest (N 58°08.456’, E 11°51.372′ trap ID 31, coll. ID 1062, 2004.viii.11-2004.viii.22); 2 females and 1 male from Sweden, Småland, Nybro kommun, Bäckebo, deciduous forest (N 56°55.299′ E 16°6.074′ trap ID 1000, coll. ID 1323, 2005.vii.02-2005.vii.12) and finally 2 females and 2 males from Sweden, Småland, Söderåkra, Påboda, garden (N 56°26.080′ E 16°4.236′ trap ID 2046, coll. ID 2053, 2008.vi.15-2008.vii.01).

Specimen parts studied with SEM were macerated to remove the soft internal tissues in a 10% KOH solution, either by leaving them in the solution for one or two days in room temperature, or by carefully boiling them in the solution for approximately ten minutes. The parts were then cleaned in water, a series of increasing EtOH dilutions, and finally in concentrated ammonia (10% KOH -> H_2_O -> 20% EtOH -> 50% EtOH -> 70% EtOH -> 90% EtOH -> 95% EtOH -> pure NH3) for at least 10 minutes in every solution except the last, in which the ammonia was just allowed to evaporate. The parts were then gold-coated and studied in a Philips XL30 Scanning Electron Microscope. The SEM micrographs were edited by blotting out obvious dirt particles or other artifacts using Adobe Photoshop CS3. Line drawings were produced in Adobe Photoshop based on SEM micrographs. Color pictures of specimen parts were photographed with a Leica MZ16 light microscope equipped with a Leica DFC420 camera. These parts were kept in an ethanol bath and illuminated from beneath. These pictures were also edited using Adobe Photoshop CS3, as described above.

## Results

The antennae are described as if they were directed strictly forwards. The legs are described as if the coxae were directed strictly downwards and the rest of the legs were extended in a right angle from the body. For the legs distal to the coxae, the preaxial surface is then anterior, the postaxial surface posterior, the outer surface dorsal and the inner surface ventral.

Terminology of surface sculpturing and of exoskeletal structures follows “The Torre-Bueno Glossary of Entomology” [64] and Ronquist and Nordlander [25], with additions from Alam [5], [6], Richards [19], Harris [65], Gibson [30], [32], Schulmeister [39] and Vilhelmsen et al. [66]. Abbreviations and naming of the wing veins and the naming of the wing cells follow Wharton et al. [2], a system in which the naming of the wing veins is based on the classical works by Comstock and Needham [67], [68], while the wing cells are given descriptive names that are not tied to wing veins. Flagellomeres are abbreviated F1 for the first flagellomere (excluding the annellus), F2 for the second, etc. The abdominal terga and sterna are abbreviated T2 for the second abdominal tergum (the petiolar tergum) and S2 for the second abdominal sternum (the petiolar sternum), etc.

The introductory and more detailed descriptions refer to *Opius dissitus*. The morphology of *Biosteres carbonarius* is described at the end of each section and only when it differs substantially from the former. Additional illustrations of *O. dissitus* (Morphbank ID 999019393) and *B. carbonarius* (Morphbank ID 999019395) appear on Morphbank (http://www.morphbank.net). New terms or definitions proposed in the text, as well as the potentially controversial choices we have made among existing terms, are discussed in the terminology section below.

*O. dissitus* is a small wasp of about 1.3 mm in body length, whilst *B. carbonarius* is about 4.0 mm in body length and noticeably more sculptured.

### Head

#### Opius dissitus

**Figure 1:**
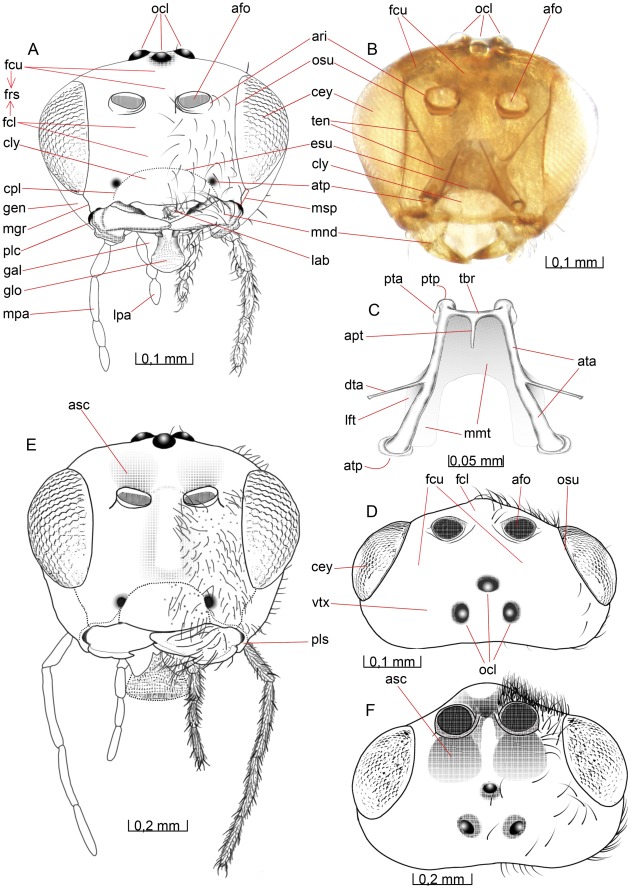
Details of the head. 1A–D *Opius dissitus* Muesebeck, 1E–F *Biosteres carbonarius* Nees. (A) Head, anterior view. (B) Head, anterior view. (C) Tentorium, dorsal view. (D) Head, dorsal view. (E) Head, anterior view. (F) Head, dorsal view.

**Figure 2:**
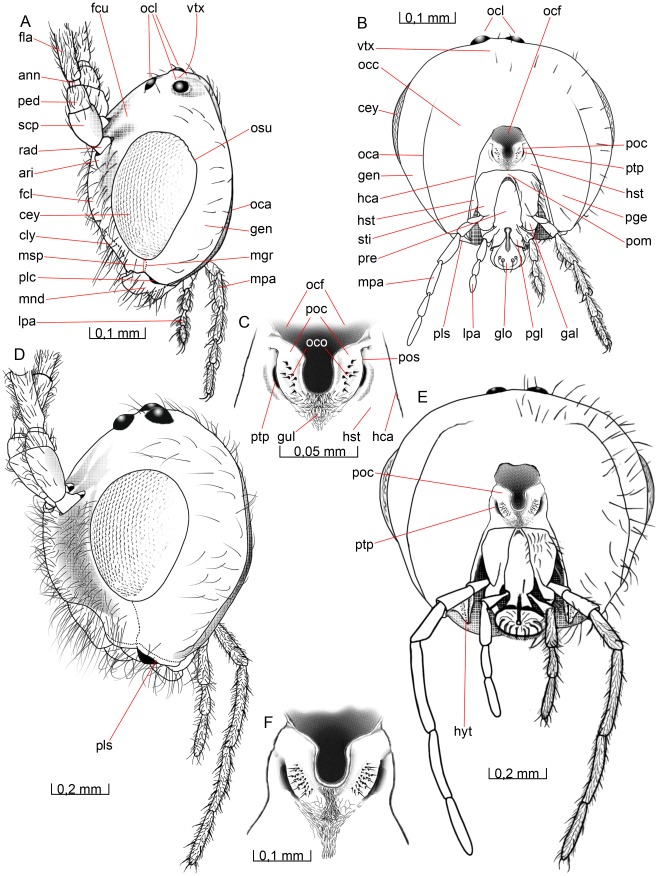
Details of the head. 2A–C *O. dissitus*, 2D–F *B. carbonarius*. (A) Head, lateral view. (B) Head, posterior view. (C) Details of the posterior part of the head. (D) Head, lateral view. (E) Head, posterior view. (F) Details of the posterior part of the head.

The **cranium** (Figs. 1A–B, 1D–F, 2A–B, 2D–E) is about 1.5 times wider than high in anterior view. In lateral view it is “D-shaped”, that is, domed anteriorly and flat posteriorly (difficult to see in Fig. 2A).

The **vertex** (vtx Figs. 1D, 2A–B) is smooth and glabrous except for a few setae around its edges. The three **ocelli** (ocl Figs. 1A–B, 1D, 2A–B) are arranged in an equilateral triangle at the top of the head capsule and the distances between them are almost equivalent to their diameter. The distance between each posterior **ocellus** and nearest **compound** **eye** (cey Figs. 1A–B, 1D, 2A–B) is equal to the length of each of the sides of the **ocellar triangle**.

The smooth **upper face** (fcu Figs. 1A–B, 1D, 2A) is glabrous except for the ventral edge, around the **antennal foramen** (afo Figs. 1A–B, 1D), where it is sparsely pubescent. There is a shallow **antennal scrobe** (asc Figs. 1E–F) above each of the antennal foramina. The diameter of each antennal foramen slightly exceeds the distance between them (∼1.25x) and is almost twice (∼1.7x) the distance between the compound eyes and the elevated and slightly strengthened **antennal rim** (ari Figs. 1A–B, 2A).

The **lower face** (fcl Figs. 1A, 2A) is just about three times as wide as high, almost flat, sparsely setiferous laterally, glabrous and somewhat convex medially (Fig. 1B). The dark brown compound eyes (cey Figs. 1A–B, 1D, 2A–B) extend just slightly from the cranial capsule even though they are relatively big; the distance between them slightly exceeds their height. They are almost twice as high as wide and the distance between their posterior margin and the occipital carina (see below) is about half the compound eye width. The compound eyes are surrounded by a vague **ocular suture** (osu Figs. 1A, 1D, 2A). About ten small **interommatidial setae** are unevenly scattered between some of the ommatidia in the central part of the eyes.

The sparsely pubescent, brown-yellowish **clypeus** (cly Figs. 1A–B, 2A) is more than twice as wide as high and delimited from the face dorsally by the **epistomal sulcus** (esu Figs. 1A–B) and laterally by the **clypeo-pleurostomal lines** (cpl Fig. 1A). Its otherwise straight ventral margin has a small tooth-like process on each side, close to its lateral edge. Between the ventral margin of the clypeus and the dorsal surface of the mandibles is a semi-circular or elliptic gap to be found when the mandibles not is completely closed, creating a “subcyclostomic” appearance. The **anterior tentorial pits** (atp Figs. 1A–C) separate the epistomal sulcus on each side from the clypeo-pleurostomal lines and mark the external invagination of the tentorium (see below).

The **gena** (gen Figs. 1A, 2A–B) is posterolaterally smoothly and evenly arched and covered with only a few setae. The **malar space** (msp Figs. 1A, 2A) is comparatively narrow; the length of the **malar groove** (mgr Figs. 1A, 2A) dividing the gena from the frons is just about 1/5 of the height of the eye.

The **pleurostoma** (pls Figs. 1E, 2B, 2D) is posteriorly united with the **hypostoma** (hst Figs. 2B–C), laterally with the gena and anteriorly with the lower part of the face and serves as the attachment for the mandibles (see below). The pleurostoma is not defined from the gena or the hypostoma by any external line or carina. The hypostoma is distally demarcated by the **hypostomal carina** (hca Figs. 2B–C) on the posterior part of the cranium and its ventral inflected margin serves as the attachment for the remaining mouthparts.

The **pleurostomal condyle** is the anterior (dorsal) of the two mandibular articulation points of the cranium and lies just posteroventrally to the **pleurostomal carina** (plc Figs. 1A, 2A), right between the ventral ends of the clypeo-pleurostomal lines (cpl Fig. 1A) and malar groove. The **posterior mandibular articulation** of the cranium is the acetabulum that is to be found between the ventral ends of the occipital carina and the hypostomal carina.

The **occiput** (occ Fig. 2B) is surrounded by sparsely scattered setae but otherwise it is glabrous except for one or two setae in its ventral region, the **postgena** (pge Fig. 2B). Its outer margin is defined by an **occipital carina** (oca Figs. 2A–B), which is present laterally but absent dorsally so that the upper ends of each lateral section of the carina are separated by a distance equal to the length of each lateral carina. Ventrally the occipital carina is clearly separated from the hypostomal carina and meets the pleurostomal carina at the base of the mandible.

The **occipital foramen** (ocf Figs. 2B–C) is the central hole in the back of the head. It is through this opening that the tracheae, nerves, muscles and other internal structures of the **cervix** pass between the **head** and the **mesosoma**. The occipital foramen is dorsally surrounded by the occiput, which is not thickened or raised to form any strengthened or specialized structure along the margin of the foramen. The ventral part of the foramen is surrounded by the **postocciput** (poc Figs. 2B–C, 2E), which is laterally set off from the remaining occipital arch by a faintly outlined **postoccipital suture** (pos Fig. 2C) and ventrally by a diffuse, subtriangular and strongly but minutely wrinkled and densely pubescent **gula** (gul Fig. 2C).

The distinct, slot-like **posterior tentorial pits** (ptp Figs. 1C, 2B–C, 2E) are located dorsally on the postoccipital suture. More than 2/3 of the postocciput, between its dorsal part and the gula, is occupied by the **occipital condyle** (oco Fig. 2C) of each side, the processes onto which the head articulates with the cervical prominence (see below) of mesosoma. The occipital condyle is covered with a hair patch consisting of about ten short, stout setae scattered evenly over the central area of the process.

The anterior tentorial pits (atp Figs. 1A–C) and posterior tentorial pits (ptp Figs. 1C, 2B–C, 2E) mark the four points where the exoskeleton is invaginated to form the internal skeletal structure of the head, the **tentorium** (Fig. 1C, ten Fig. 1B). The circular anterior tentorial pit is the anterior attachment for the **anterior tentorial arm** (ata Fig. 1C) while the elongated posterior tentorial pit is the attachment for the **posterior tentorial arm** (pts Fig. 1C). The tentorium is hollow, but a small membrane inside each of the tentorial pits seals the interior off from the exterior.

The anterior tentorial arm is thickest at its anterior end and bends initially towards the anterior tentorial arm of the other side, while its posterior half runs almost parallel to the arm of the other side. The much thinner and apically tapering **dorsal tentorial arm** (dta Fig. 1C) arises from the dorsal surface of the anterior tentorial arm, slightly anterior to the middle of the latter, and is directed towards the cranium, where it attaches close to the ocular suture (osu Figs. 1A, 1D, 2A), in height with, but well separated from, the antennal foramen (afo Figs. 1A–B, 1D). In contrast to the attachment of the other tentorial arms, those of the dorsal tentorial arms leave no external trace on the cranium. A U-shaped **mesal membrane of the anterior tentorial arm** (mmt Fig. 1C) is attached anteroventrally along the anterior tentorial arm. The membranes together cover more than two thirds of the space between the anterior tentorial arms. A semi-circular **lateral flag of the anterior tentorial arm** (lft Fig. 1C) covers almost one third of the length of the arm. The flag membrane is widest close to the point of attachment of the dorsal tentorial arm, where it is slightly wider than the anterior tentorial arm itself.

The posterior tentorial arm is about half as long as it is wide, and it is shaped more like an invaginated rim than an arm or a bar. Its posteroventral edge is drawn out into a process close to the ventral margin of the occipital foramen. The process-like formations on each side almost reach each other (not depicted here), forming a soft transition between the posterior tentorial arm and the reinforced lateroventral margin of the postocciput. The left and the right sides of the tentorium are connected to each other through the **tentorial bridge** (tbr Fig. 1C), just where the anterior and the posterior tentorial arms meet each other, close to the posterior end of the tentorium. The tentorial bridge is about half as thick as the anterior and posterior tentorial arms, where these meet. In the middle of the tentorial bridge, there is an anteriorly directed and tapering **anterior process of the tentorial bridge** (apt Fig. 1C). This process is of about the same length as the tentorial bridge, and serves as the tendon for a contractor muscle of the **pharynx** (not depicted here).

#### Biosteres carbonarius

The cranium in *B. carbonarius* is more rigid than in *O. dissitus* and the frons and clypeus are more densely pubescent. The cranium is also much more quadratic in anterior view, i.e. it is only slightly wider than high. The compound eye is just slightly higher than wide and the shortest distance between the compound eye and the occipital carina is about the same length as the width of the eye. Interommatidial setae are apparently lacking.

The clypeus is both more setose and punctuated. It is less than twice as high as wide and its smoothly bent ventral margin, without tooth-like processes, leaves no gap or orifice towards the mandibles. The malar space is much wider than in *O. dissitus*, about half the height of the eye. *B. carbonarius* has shallow but slightly more distinct antennal scrobes. There is also a prominent wedge-shaped, mostly glabrous crest from between the antennal sockets and almost all the way down to the epistomal sulcus (esu Figs. 1A–B). The pleurostoma is very narrow and demarked by a faint but still detectable line around its entire edge, except for where it meets the hypostomal carina (hca Figs. 2B–C), where it is drawn out into a ventrally protruding **hypostomal tooth** (hyt Fig. 2E).

The smooth occiput has some scattered setae also mesad to the occipital carina. The dorsal ends of the occipital carinae of each side are situated much closer to each other than in *O. dissitus* and the terminal parts of the carinae are somewhat more irregular. Ventrally, the occipital carina is well separated from the hypostomal carina. The occipital condyle (oco Fig. 2C) occupies about half of the postocciput (poc Figs. 2B–C, 2E) and is covered with about twenty setae. The gula (gul Fig. 2C) is narrower and extends farther ventrally on the posterior surface of the cranium. The tentorial bridge in *B. carbonarius* is thicker than in *O. dissitus*, almost as thick as the anterior tentorial arm.

### Antennae

#### Opius dissitus

**Figure 3:**
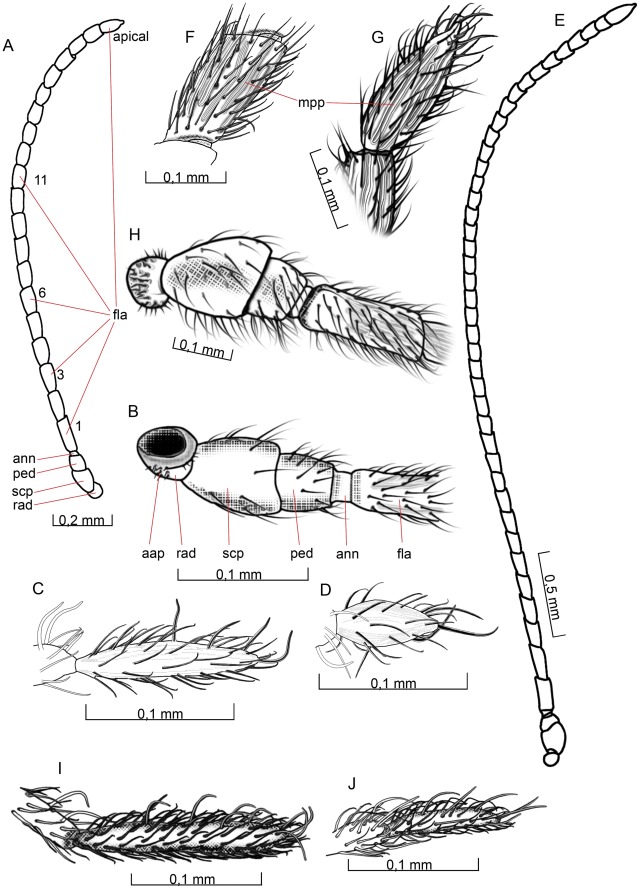
Details of the head. 3A–D *O. dissitus*, 3E–J *B. carbonarius*. (A) Antennae. (B) Basal antennal articles, posterior (inner) view. (C) Apical maxillary palpomere. (D) Apical labial palpomere. (E) Antennae. (F) Third flagellomere. (G) Apical flagellomere. (H) Basal antennal articles, anterior (outer) view. (I) Apical maxillary palpomere. (J) Apical labial palpomere.

The dark, threadlike (filiform) **antenna** has 19 – 24 **articles** (i.e. 17 – 22 flagellomeres). There is no difference between the sexes in the number of flagellomeres. The first segment is the yellowish **scape** (scp Figs. 2A, 3A, 3B), which is sparsely covered with about 15 setae on the inner surface but almost glabrous on the outer surface. The basal part of the scape is the bulb-shaped **radicle** (rad Figs. 2A, 3A–B), which is mainly hidden in the antennal foramen (afo Figs. 1A–B, 1D). It is defined by a strong constriction, but it does not articulate with the remaining part of the scape. The radicle is equipped with approximately 35 short, stout mechanosensory setae and has a basal, condyle-like process, the **antennal articular** process (aap Fig. 3B, but not obviously seen here), which articulates with the **antennifer** of the antennal rim (ari Figs. 1A–B, 2A). Distad of the constriction, the scape is shaped like an eggcup. Distally it is about as broad as the length from the constriction to the moderately bent distal edge.

**Figure 4:**
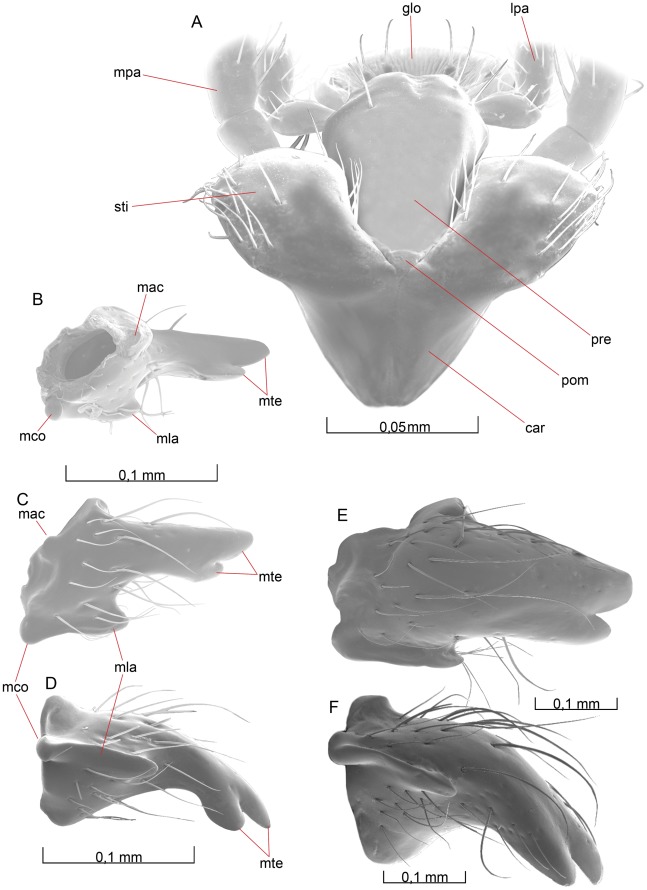
Details of the head. 4A–D *O. dissitus*, 4E–F *B. carbonarius*. (A) Labiomaxillary complex, posteroventral view. (B) Mandible, laterodorsal view. (C) Mandible, anteroventral view. (D) Mandible, ventral view. (E) Mandible, anterior view. (F) Mandible, ventral view.

The second antennal segment, the **pedicel** (ped Figs. 2A, 3A–B), is dark, slightly cone-shaped and about as long as its basal width. It is more densely setose than the scape with about 30 setae (set Fig. 4C–D). The short, ring-like yellow **annellus** (ann Figs. 2A, 3A–B) is glabrous. The **flagellomeres** (fla Figs. 2A, 3A–B) become progressively shorter distally except for the last one. **F1** is approximately three times as long as wide and the subapical flagellomere is just slightly more than 1.5 times long as broad. The cone-shaped, apical flagellomere is almost three times as long as its subbasal width and is drawn out apically into a nipple-like structure.

On each of the flagellomeres there are approximately 90–100 setae and about eight elongated **multiporous plates** (mpp Figs. 3F–G) that are more or less as long as the individual flagellomeres. On the most apical flagellomere, however, the multiporous plates extend from the base to only about two thirds of the flagellomere length. The annellus completely lacks multiporous plates.

#### Biosteres carbonarius

The thread-like antenna of *B. carbonarius* is densely setose. It is noticeably longer than the fore wing and had at least 45 articles in all dissected specimens. The scape, pedicel and annellus are dirt-yellowish like the basal third of the first flagellomere. The remainder of the antenna is black. The first flagellomere (F1) is about 2.5 times as long as wide. The first eight basal flagellomeres become progressively somewhat shorter whilst the remaining flagellomeres are of about the same length, except for the much longer, cone-shaped apical flagellomere.

### Mouthparts

#### Mandible

##### Opius dissitus

The **mandible** (Figs. 4B–D, mnd Figs. 1A–B, 2A) is brown-yellowish. The basal half is sparsely covered with about 20 medium to long setae, evenly spread around both the inner and outer sides of the mandible. The basal part of the mandible is strongly expanded ventrally and equipped with a prominent **mandibular lancea** (mla Figs. 4B–D). Each mandible has two attachment points to the cranium: the ventrolateral **mandibular condyle** (mco Figs. 4B–D), which fits into the **pleurostomal acetabulum**, and the dorsolateral **mandibular acetabulum** (mac Figs. 4B–C), which receives the **pleurostomal condyle**, in anterior view hidden by the ventral rim of the frons.

Distal to the mandibular lancea, the mandible is distinctly constricted and glabrous. From that point, the mandible expands somewhat distally and ends in two apical **mandibular teeth** (mte Figs. 4B–4D). The upper tooth is a straight continuation of the dorsolateral edge of the mandible and reaches a bit longer than the second tooth. The latter can be hard to see without dissection as it is typically hidden behind the more apical tooth in normal repose.

##### Biosteres carbonarius

The mandible in *Biosteres* (Figs. 4E–F) is noticeably more robust than in most other opiines. In *B. carbonarius*, its color is brown-yellowish with black apical teeth and it is more densely setose than the mandible of *O. dissitus*. The basal part of the mandible is only slightly expanded ventrally but is nevertheless equipped with a distinct mandibular lancea. The distal half of the mandible remains almost evenly broad all the way out to the two equally long apical mandibular teeth.

#### Labrum and labiomaxillary complex

##### Opius dissitus

The **labrum** (lab Fig. 1A) is flat and smooth with its ventral and lateral margins abundantly pubescent. It has a distinctly arched ventral margin.

The proximal part of the maxilla is the **cardo** (car Fig. 4A). In the two species studied here the two cardines are fused, creating a unit which in normal repose is hidden behind the more distal, white-yellowish, somewhat kidney-shaped, smooth and unsculptured **stipes** (sti Figs. 2B, 4A). The ventrobasal and ventrolateral edges of the stipes are sparsely covered with approximately 20 setae.

The white-yellowish six-segmented **maxillary palp** (mpa Figs. 1A, 2A–B, 4A) is about two thirds of the width of the head and articulates basally with the apicolateral area of the stipes. Its first segment is almost glabrous apart from two proximal setae. The second segment is covered with 8±2 setae proximally, whilst the four following segments have approximately 50±5 setae each. Among the setae on the four apical segments, there are three or four on the distal half that are distinctly thicker and longer than the others. The two proximal segments combined are as long as each one of the remaining four segments, which are approximately of equal length. The last segment of the maxillary palp (Fig. 3C) is roughly five times as long as broad and it is apically equipped with one markedly long and thick seta.

The brown-yellowish **lacinia** (not illustrated) and **galea** (gal Figs. 1A, 2B) are attached to the apical part of the stipes. The lacinia is a thin but hard, sail-like sclerite, hidden between the galea and the **prementum** (pre Figs. 2B, 4A). The apicoventral margin of the lacinia is densely set with short, stout setae. The basal part of the galea is a cone-like and rigid structure, supporting the thinner but heavily sclerotized lateroventral lobe of the galea, which covers the softer mouthparts beneath it. Each galea is sparsely set with about 20±5 rather long setae, most of them on the apical lobe while the basal and thicker part of the galea is almost glabrous. The apical margin of the galea is densely covered with short, stout setae.

The white-yellowish, mostly weakly sclerotized **labium** forms the innermost core and posterior wall of the **labiomaxillary complex**. The glabrous and minute **postmentum** (pom Figs. 2B, 4A) is the most proximal part of the **labium**, situated between the basal parts of the stipites. It is a very small, triangular, sclerotized plate with softer and flexible membranous sides connecting it to the surrounding parts. The postmentum is followed distally by the prementum, which is somewhat tulipiform in posterior view. The prementum is entirely smooth and glabrous with the exception for two subapical setae on each side, adjacent to the membranous attachments of the labial palpi on the posterolateral margin of the prementum. The **labial palp** (lpa Figs. 1A, 2A–B, 4A) is four-segmented.

All but the second of the four segments of the labial palp are approximately of the same length; about half the length of one of the four distal segments of the **maxilla**. The basal segment widens distally, and on its apical third it is equipped with approximately 10 setae. The second labial segment is about 30% longer than the first. It is cylindrical and slightly more than twice as long as wide, equipped with about 22±2 setae evenly scattered over its surface except for its glabrous ventral [inner] and basal fourth. The third segment widens distally and is evenly covered with 20±2 setae, whilst the fourth segment is more cylindrical. It is evenly covered by 35±5 setae and its length is almost twice its width. The three last segments of the labial palp have two, three and five distinctly thicker and longer, fluted setae on their posterior, distal half. The most obvious of these setae is situated at the apex of the last segment of the labial palp.

The terminal lobe of the labium is the **glossa** (glo Figs. 1A, 2B, 4A), the membranous ventral surface of which is wrinkled and equipped with four robust setae subapically whilst its dorsal surface is distally equipped with 6 – 8 serrated bands. The membranous **paraglossa** (pgl Fig. 2B) is glabrous on its posterior [outer] surface but densely covered with a huge number of very small ligulate hairs on its anterior [inner] surface.

##### Biosteres carbonarius

The maxillary palp is distinctly longer than in *O. dissitus*, almost as long as the width of the head. Its first cone-shaped segment is glabrous. The distal half of the second segment is proximally richly setose, whilst the remaining four apical segments are all densely setose. As in *O. dissitus*, these four apical segments are equipped with some noticeably longer, fluted setae, but in *B. carbonarius* they are more numerous. Segment four and five are considerably longer than the other segments of the maxillary palp, and of these two, segment four is the longest. The apical maxillary segment (Fig. 3J) is more than six times as long as thick.

The apical segment of the labial palp is more drawn out into a cylindrical shape in *B. carbonarius* than in *O. dissitus*.

### Mesosoma

**Figure 5:**
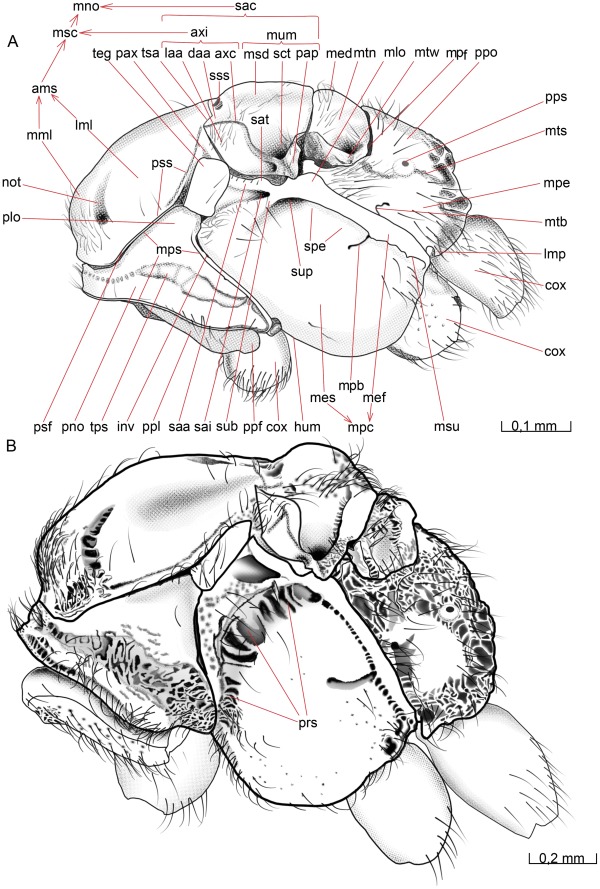
Mesosoma, lateral view. 5A *O. dissitus*, 5B *B. carbonarius*.

**Figure 6:**
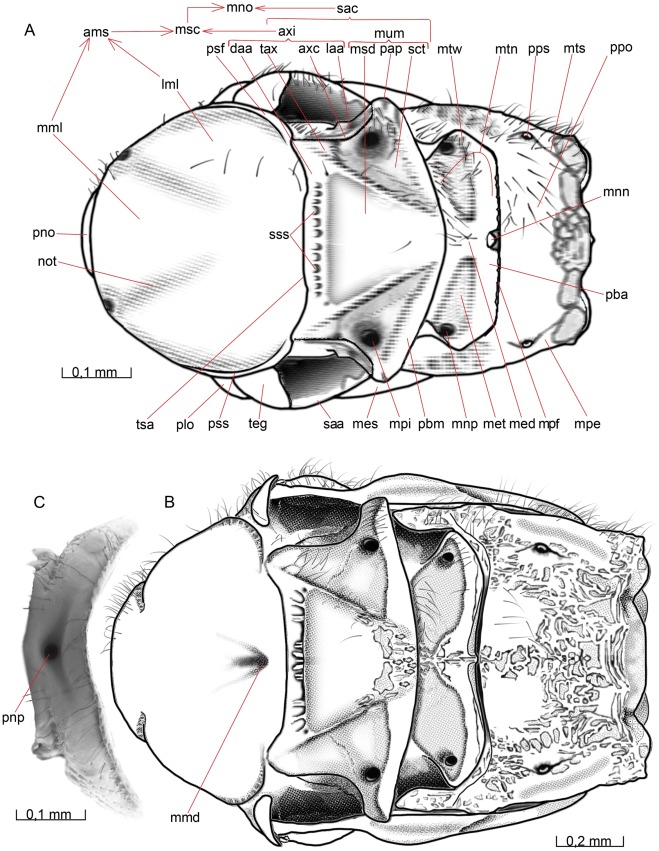
Mesosoma, dorsal view. (A) *O. dissitus*. (6B) *B. carbonarius*. (C) Pronotum of the latter.

The mesosoma of *O. dissitus* is all black (except the yellowish **tegulae** (teg Figs. 5A, 6A), rather arched and about 1.5 times as long as high and in dorsal view approximately 25% narrower than the head. The mesosoma of *B. carbonarius* is generally more sculptured than that of *O. dissitus*.

#### Pronotum

##### Opius dissitus

**Figure 7:**
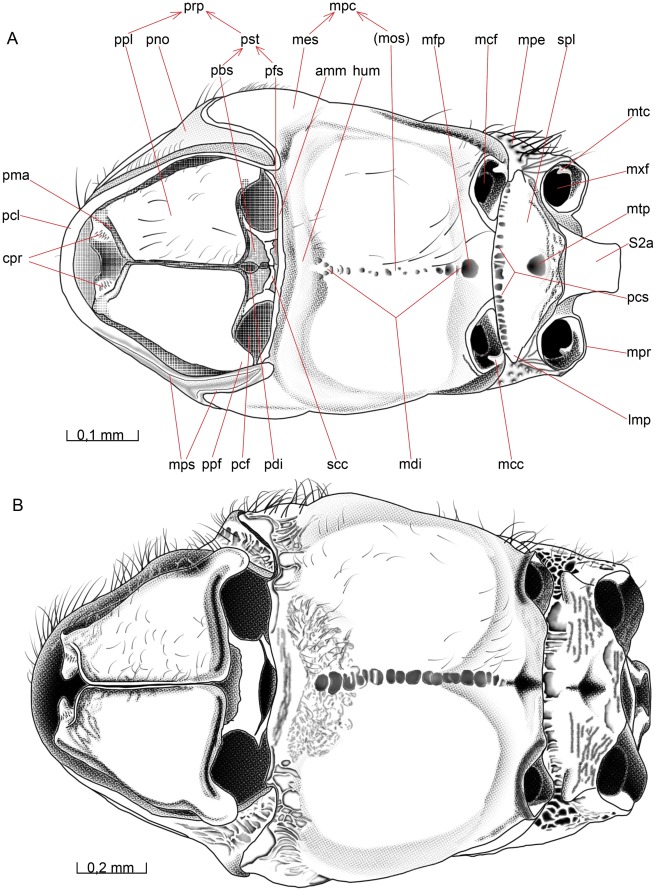
Mesosoma, ventral view. 7A *O. dissitus*. 7B *B. carbonarius*.

**Figure 8:**
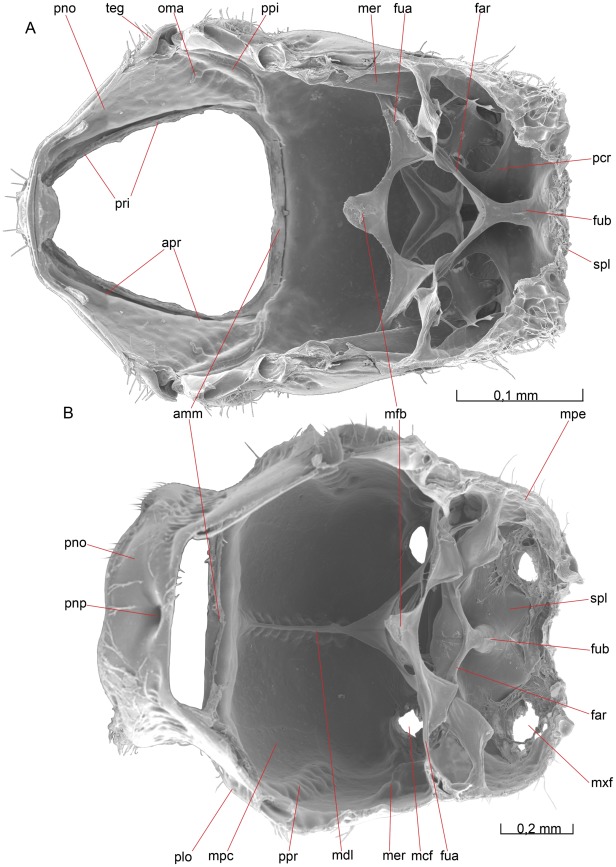
Mesosoma, internal view. 8A–B *B. carbonarius*. (A) Ventral view. (B) Anteroventral view.

**Figure 9:**
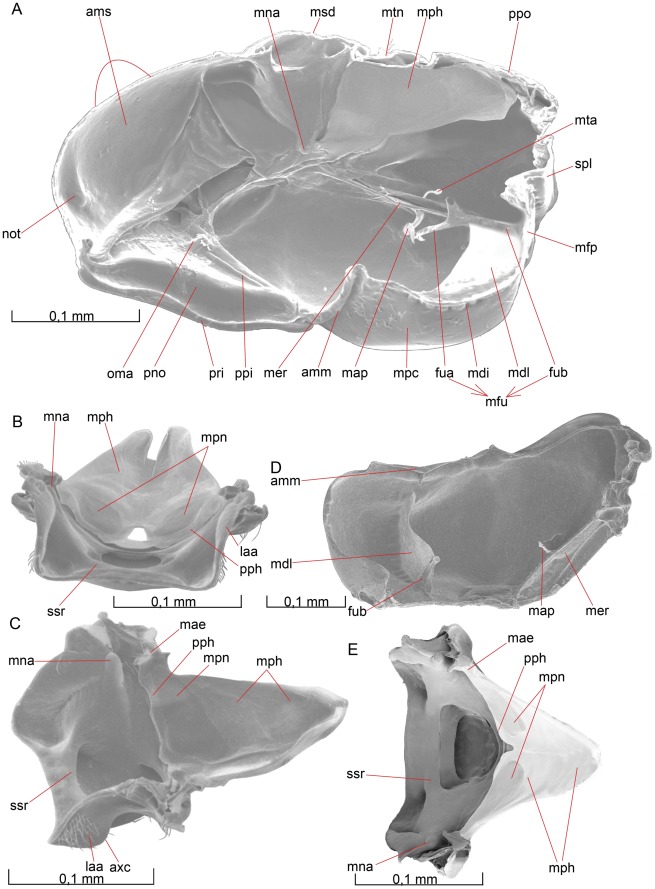
Details of mesosoma. 9A–D *O. dissitus*, 10E *B. carbonarius*. (A) Anteroventral, lateral internal view. (B) Mesonotum and mesopostnotum, anteroventral internal view. (C) Mesonotum and mesopostnotum, venterolateral view. (D) Mesopectus, internal posterodorsal view. Left mesopleuron removed. (E) Mesonotum and mesopostnotum, ventral view.

The **pronotum** (pno Figs. 5A, 6A, 7A, 8A–B, 9A) is glabrous except for a row of 10–15 setae along its anterolateral margin. It consists of two, big subtriangular lateral parts connected to each other dorsally by a collar-like median band. A narrow **marginal pronotal sulcus** (mps Figs. 5A, 7A) runs along the entire tergites outer margin, except for an interruption at the posterodorsal, flap-like **pronotal lobes** (plo Figs. 5A, 6A, 8B). This sulcus is the landmark for the **pronotal inflection** (pri Figs. 8A, 9A), which serves as a strengthened rim of the sclerite’s anteroventral edge, i.e. towards the head and propleura. Posterodorsally, the rim is raised into a ridge, the **posterior pronotal inflection** (ppi, Figs. 8A, 9A), which serves as an interior locking mechanism between the pronotum and the anterior margin of the mesonotum The pronotal lobe covers the **anterior thoracic spiracle**. Beneath the pronotal lobe, at the posterior margin of the pronotum, is a small **invagination for the occlusor muscle apodeme** (inv Fig. 5A). This small pit marks the **occlusor** **muscle apodeme of anterior thoracic spiracle** (oma Fig. 8A, 9A), which is directed anteriorly from the posterior pronotal inflection.

The smoothly rounded posteroventral corner of the pronotum reaches slightly beyond the posterolateral edge of the **procoxal foramen** (pcf Fig. 7A). The posteroventral corners of the pronotum are separated from each other ventrally by a distance corresponding to slightly less than the maximum width of the ventral cavity defined by the pronotum. From the posteroventral corner of the pronotum runs a vague, lacunated **transverse pronotal sulcus** (tps Figs. 5A) across the pronotum to the other posteroventral corner. The transverse pronotal sulcus is as widest just below the height of the pronotal lobe, where it is about a third of the width of the lateral part of the pronotum. Dorsomedially, the transverse pronotal sulcus is more foveated, the central fovea being enlarged to form a small but distinct **pronope** (pnp Figs. 6C, 8B). Internally, the sulcus corresponds to smooth **anteromedian pronotal ridge** (apr Figs. 8A–B).

##### Biosteres carbonarius

The pronotum is heavily sculptured with a deep and conspicuous pronope (pnp Figs. 6C, 8B). The dorsolateral surface of the transverse pronotal sulcus, including the pronotal lobe, is glabrous except for a few scattered setae and a row of setae along the posterodorsal edge. The anterolateral and anterodorsal (i.e. the pronotal collar) areas of the pronotum are pubescent.

#### Propectus

##### Opius dissitus

The **propectus** (prp Fig. 7A) consists of the two propleura and the prosternum. They are distinct sclerites but form a tightly integrated unit, which is rather movable in relation to the remaining mesosoma. The propectus serves as the attachment point for the head and fore legs.

#### Propleuron

##### Opius dissitus

**Figure 10:**
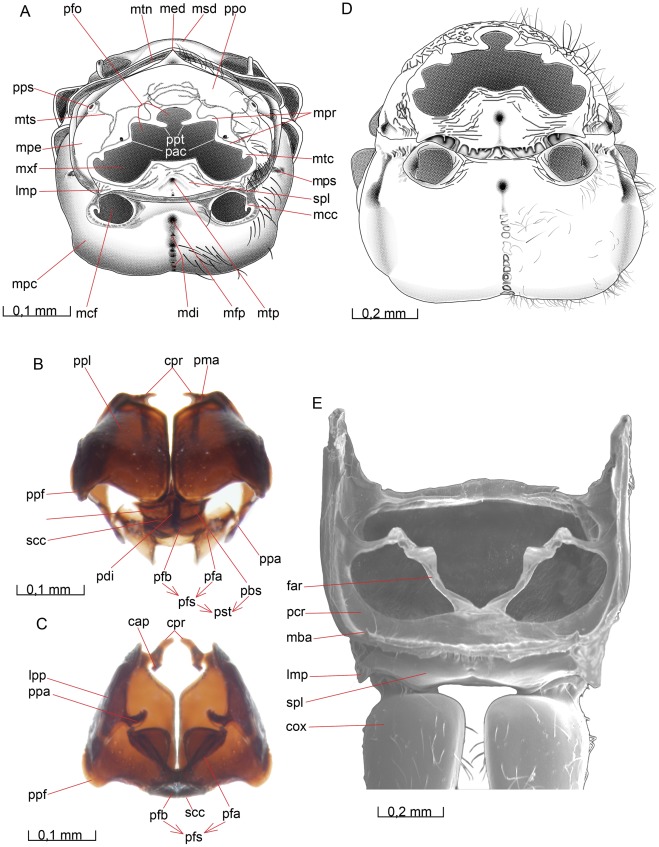
Details of mesosoma. 10A–C, E *O. dissitus*, 10D *B. carbonarius*. (A) Mesosoma, posterior view. (B) Propectus, posteroventral view. (C) Propectus, posterodorsal view. (D) Mesosoma, posterior view. (E) Metapectal-propodeal complex, anterior (internal) view.

The lateral and dorsal surfaces of the **propleuron** (ppl Figs. 5A, 7A, 10B) are hidden inside the thorax; they constitute about one fourth of the total propleural area. The external surface of the propleuron is almost glabrous and sparsely covered with about 35–40 scattered setae. The setae are denser towards the mesal and lateral edges but the propleuron is almost glabrous towards the apical and posterior edges. The mesal surface of the propleuron is equipped anteriorly with a down-curving, flap-like anteroventral process, the **cervical prominence** (cpr Figs. 7A, 10B–C), which serves as the articulation point for the occipital condyle (oco Fig. 2C) of the head. Each cervical prominence is furnished preapically with a hair patch consisting of about 20–25 short setae scattered evenly over the lateral surface of the ball-like apex. The cervical prominence is equipped posteriorly with a **cervical apodeme** (cap Fig. 10C) which is the site of insertion of laterocervical muscles.

The anterior edge of the propleuron is strengthened by a rim, the **propleural marginal area** (pma Figs. 7A, 10B), posteriorly delimited by a delicate line. The area is generally narrow but widens slightly anterolaterally. The propleura abut medioventrally but they are not merged. The membrane separating the propleura is wide enough for the sclerites to move independently to some extent. The lateral margin of the ventral surface of the propleuron is evenly bent in a smooth bow, ending in a glabrous **propleural flange** (ppf Figs. 5A, 7A, 10B–C), a subtriangular flap-like extension ventrally at the postero-lateral corner of the propleuron. The outer lateral area of the propleura are dorsally inflected, forming an obliquely vertical surface, the **lateral propleural area** (lpp Fig. 10C) that is drawn out posteromesally, ending in the **posterior** **propleural arm** (ppa Figs. 10B–C). This apodeme serves as the site of insertion of the propleuro-pronotal muscles but is also the attachment point for the lateral end-knob of the profurcal arm.

##### Biosteres carbonarius

Except for the propleural flange, the propleuron is covered with scattered setae. The anterior and ventromedial margins are more or less wrinkled but the rest of the pleuron is smooth. The lateral margin of the ventral surface is somewhat sinuate, ending posteriorly in a smooth, rounded, almost waist-like constriction at the base of the propleural flange. The entire margin of the ventral surface of the propleuron is marked by a narrow furrow, outside of which there is a distinct, strengthened rim.

#### Prosternum

##### Opius dissitus

The **prosternum** (pst Fig. 7A, 10B) is externally subdivided by a transverse **sternacostal carina** (scc Figs. 7A, 10B–C) into an anterior horizontal, trapezoidal **probasisternum** (pbs Figs. 7A, 10B) and a posterior, vertically oriented **profurcasternum** (pfs Figs. 7A, 10B–C). The latter is largely an internal apophysis complex consisting of a basal **profurcal base** (pfb Figs. 10B–C), with a reinforced edge, and two elongated **profurcal arms** (pfa Figs. 10B–C), each bearing a comparatively large membranous flange and a lateral end-knob attaching to the corresponding propleural arm. The sternacostal carina is about ten times as long as wide. The posterolateral margin of the probasisternum and the lateral margin of the profurcasternum are raised to form a stout rim along the medial part of the procoxal foramen. The raised rim meets, but is distinct from, the sternacostal carina.

The basisternum is divided longitudinally by the **prodiscrimen** (pdi Figs. 7A, 10B). The anterior edge of the basisternum is drawn out to form a median process, which is covered externally by the propleura. The anterior basisternal process is visible only if the propectus is dissected.

##### Biosteres carbonarius

The sternacostal carina is much thicker than in *O. dissitus*, being only about four times as long as wide. *B. carbonarius* has no prodiscrimen in the true sense that corresponds with the discrimenal lamella; instead, the big, elliptic profurcal pit extends anteriorly to the anterior edge of the basisternum.

#### Mesonotum

##### Opius dissitus

An uninterrupted and distinct **transscutal articulation** (tsa Figs. 5A, 6A) divides the **mesonotum** (mno Figs. 5A, 6A) into an anterior mesonotal sclerite, the **anteromesoscutum** (ams Figs. 5A, 6A, 9A), and a posterior **scutellar-axillar complex** (sac Figs. 5A, 6A), which includes the posterolateral parts of the **mesoscutum** (msc Figs. 5A, 6A), i.e. the **axillae** (axi Figs. 5A, 6A), as well as the **mesoscutellum** (mum Fig. 5A, 6A).

#### Anteromesoscutum

The anteromesoscutum (ams Figs. 5A, 6A, 9A) is mostly glabrous except for the sparsely pubescent anterior fifth and a few setae along its lateral margins, a few setae on the **preaxilla** (pax Fig. 5A) and a sparse row of setae posterolateral to the faint **notauli** (not Figs. 5A, 6A, 9A).

Submarginally, along the mesonotal lateral margin, runs an obscure **parascutal sulcus** (pss Figs. 5A, 6A), separating an indistinct, narrow and glabrous **parascutal flange** (psf Figs. 5A, 6A) from the remainder of the mesoscutum. The parascutal sulcus is missing anteriorly, the missing part being about as long as each of the lateral sections of the sulcus. Furthermore, the parascutal sulcus consists of a posterior, slightly more dorsal section and an anterior, slightly more ventral section, which do not quite meet but run in parallel above the pronotal lobe (plo Figs. 5A, 6A, 8B) and tegula (teg Figs. 5A, 6A, 8A) for a short distance. The posterior section of the sulcus is somewhat smoother and wider than the anterior section. Beneath the posterolateral part of the parascutal flange, there is a large vertical area, the preaxilla, which is in normal repose largely covered externally by the mesopectus (see below)**.** The preaxilla articulates anteriorly with the yellowish, semi-sclerotized tegula. The tegula covers the base of the fore wing and is equipped with approximately two setae posteriorly on its dorsal surface.

The notauli (not Figs. 5A, 6A, 9A) divide the mesoscutum into one **median** **mesoscutal lobe** (mml Figs. 5A, 6A) and two **lateral mesoscutal lobes** (lml Figs. 5A, 6A). The notauli begin anteriorly as lacunated impressions and fade out well before the transscutal articulation. There is no median mesoscutal depression or pit.

##### Biosteres carbonarius

In *B. carbonarius*, the parascutal flange is larger and more clearly delineated than in *O. dissitus* and it is coarsely sculptured. The notauli are broad, deep and lacunose anteriorly. They reach about one third of the length of the mesoscutum. In contrast to *O. dissitus*, there is a large and distinct ***medio-posterior mesoscutal depression*** (mmd Fig. 6B) a short distance anterior to the transscutal articulation; it appears to represent the posterior ends of the notauli judging by the direction of the latter. The scutum is heavily wrinkled around the anterior end of the notauli, but also the area between them is rather uneven and slightly hairy. The remainder of the mesoscutum is glabrous.

#### Scutellar-axillar complex

##### O. dissitus

The axilla (axi Figs. 5A, 6A) is largely glabrous with the exception for a row of setae along the posterior margin of the **triangular axillar region** (tax Fig. 6A), and about 20–25 setae on the lateral axillar area. The axilla is divided by a distinct **axillar carina** (axc Figs. 5A, 6A, 9C) into a **dorsal axillar area** (daa Figs. 5A, 6A) and a **lateral axillar area** (laa Figs. 5A, 6A, 9B–C). On the dorsal axillar area, between the **mesoscutellar pit** (mpi Fig. 6A) and the transscutal articulation (tsa Figs. 5A, 6A) and in height with the anterior edge of the **mesoscutellar disc** (msd Figs. 5A, 6A, 9A, 10A), the area is steeply bent and slopes increasingly downwards to the mesoscutellar pit.

The **scutoscutellar sulcus** (sss Figs. 5A, 6A) is composed medially of about 14, comparatively small, irregular but distinct pits and laterally ends in the mesoscutellar pit (mpi, Fig. 6A). The mesoscutellar disc (msd Figs. 5A, 6A, 9A, 10A) is subtriangular, narrowing posteriorly. It is largely glabrous with the exception for a row of setae along its lateral margin. In lateral view, the mesoscutellum is of about the same height as the mesoscutum. The posterior margin of the **mesoscutellar trough** (sct Figs. 5A, 6A) is the **posterior bar of mesoscutellum** (pbm Fig. 6A) which has the form of a somewhat strengthened and almost glabrous rim. The trough slants anterolaterally into a deep, apophysis-marking **mesoscutellar pit** (mpi Fig. 6A) and ends apically in a keel-like **postalar process** (pap Figs. 5A, 6A), which bears 15 – 20 setae dorsally.

The scutoscutellar sulcus corresponds internally to a distinct **scutoscutellar ridge** (ssr Figs. 9B–C, 9E). Together they mark the separation between the axilla (axi Figs. 5A, 6A) and the mesoscutellum. The scutoscutellar ridge is complete, while the corresponding scutoscutellar sulcus is absent laterally, leaving no visible trace between the distinct median section and the mesoscutellar pit (mpi Figure 6A). The mesoscutellar pit corresponds to a large, tubercle-shaped **mesoscutellar apodeme** (mna Figs. 9A–C, 9E).

##### Biosteres carbonarius

The mesoscutellum is more strongly raised in lateral view than in *O. dissitus*. The sculpture of the mesoscutellum varies considerably among specimens but the lateral and posterior areas are usually fairly sculptured and hairy while the dorsal area (the mesoscutellar disc) is glabrous. The median part of the scutoscutellar sulcus is comparatively broader and composed of a varying number (eight to twelve) of different-sized and irregular hollow-like pits.

#### Mesopostnotum

##### O. dissitus

The **mesopostnotum** (mpn Figs. 9B–C, 9E) is formed like a narrow transverse band equipped with a massive posterior (ventral) **mesophragma** (mph Figs. 9A–C, 9E) and a large anterior (dorsal) **pseudophragma** (pph Figs. 9B–C, 9E). The mesophragma is a large plate-like apodeme with a distinct median notch in its ventral margin for the passage of internal organs. The pseudophragma consists of two almost completely separated lateral lobes. Anterolaterally, there is a short but wide arm-like **mesopostnotal apodeme** (mae Figs. 9C, 9E) extending from the mesopostnotum towards the posterodorsal corner of the mesopectus.

##### B. carbonarius

Unlike *O. dissitus*, the dorsal margin of the pseudophragma is bowed and the anterolateral arms of the mesopostnotum are elongated and long.

#### Mesopectus

##### Opius dissitus

The **mesepimeron** is divided by a distinct **mesepimeral sulcus** (msu Figs. 5A), running parallel to and some distance in front of the posterior margin of the **mesopectus** (mpc Figs. 5A, 7A, 8B, 9A, 10A), from the middle **leg articulation** to the **subalar pit** (sup Fig. 5A) beneath the **wing articulation**. This sulcus corresponds internally to a large and strong **mesepimeral ridge** (mer Figs. 8A–B, 9A, 9D). The largely glabrous posterior area behind the mesepimeral sulcus is the **mesepimeral flange** (mef Fig. 5A). The anterior margin of the mesepimeral flange is unevenly dented, noticeably more in its lower half, beneath the **mesopleural scrobe** (mpb Figs. 5A, 10A).

The dorsal portion of the mesepimeral flange extends anteriorly as two separate reinforcements: a **subalar area** (saa Figs. 5A, 6A) that runs along the dorsal mesopectal margin and beneath that a **subalar bridge** (sub Fig. 5A), which continues onto the **mesepisternum** (mes Figs. 5A, 6A, 7A). The subalar area is equipped posteriorly with a distinct **subalar tubercle** (sat Fig. 5A), a dorsal swelling just anterior to the separation of the subalar area and the subalar bridge. Posterior to the tubercle, just before joining the subalar bridge, the subalar area is narrowed to about half its distal width. The anterior two thirds of the subalar area is equipped with five short, stout setae dorsally.

The subalar bridge is directed anteroventrally and fades out close to the anterodorsal corner of the mesepisternum. The dorsal margin of the subalar bridge is covered with about 25–30 fine, threadlike setae and its ventrolateral edge bears 5±1 considerably longer and thicker setae. The **subalar impression** (sai Fig. 5A) forms a deep pit posteriorly, at the junction between the subalar area and the subalar bridge, and becomes shallower anteriorly. The subalar impression is glabrous except for some setae anteroventrally, adjacent to the subalar bridge. The **speculum** (spe Fig. 5A) is glabrous and immaculate.

The dorsal **mesepimeral lobe** (mlo Fig. 5A) projects posteriorly to cover the posterior thoracic spiracle.

Anterior to the mesepimeral sulcus, the mesopectus is mainly glabrous except for the pubescence of the subalar bridge and subalar impression described above. The **anteroventral mesopectal margin** (amm Figs. 7A, 8A–B, 9A, 9D) is folded into a reversed, spout-like, bowed rim. The **hypocnemium** (hum Figs. 5A, 7A) is furnished with about 30 scattered setae; the epicnemial and hypocnemial carinae are missing.

The mesopleural scrobe (mpb Fig. 5A, 10A) is slightly curved and ends anteriorly in a small pit. The scrobe is also slightly more open posteriorly, where it attaches to the mesepimeral sulcus. It is approximately equal in length to the width of the mesepimeral flange (mef Fig. 5A) at the level of the fovea. Immediately beneath the junction between the dorsal epimeral lobe, the subalar bridge and the subalar area there is a big and deep, semicircular subalar pit (sup Fig. 5A). This pit marks a large internal apophysis that externally includes parts of the posterior pit of the subalar impression as well.

Lateroventrally, the mesopectus bears just a few setae except for a patch of some ten setae in front of, and dorsal to, each **mesocoxal foramen** (mcf Figs. 7A, 8B, 10A). The distance between the mesocoxal foramina is approximately 1.5 times their diameter. Each mesocoxal foramen is surrounded by a slightly elevated rim that is highest anteriorly (ventrally). A delicate line that indicates the anterior base of the raised rim runs from the epimeral flange and anterolaterally, reaching about one third of the diameter of the coxal foramen. Posterolaterally, the rim of the mesocoxal foramen passes onto the epimeron without any visible separation externally, while internally it forms the lateral **mesocoxal condyle** (mcc Figs. 7A, 10A) serving as the articulation for the mesocoxa.

The **mesodiscrimen** (mdi Figs. 7A, 9A, 10A) is a sulcus that runs along the entire ventral mid-line of the mesopectus and its irregular pits have a slight but clear tendency to become bigger posteriorly. The row ends with the distinct **mesofurcal pit** (mfp Figs. 7A, 9A, 10A) that is the external point of invagination of the **mesofurca** (mfu Fig. 9A).The mesofurca consists of a stiff and hollow **mesofurcal base** (fub Figs. 9A, 9D) and two **mesofurcal arms** (fua Figs. 8A–B, 9A). The two mesofurcal arms are connected to each other through a semi-sclerotized **mesofurcal bridge** (mfb Figs. 8A–B), that from a wide lateral attachment to each furcal arm narrows abruptly before it medially again expands and forms a semi-circular disc. The mesofurcal arm ends laterally in a tendon connected to a muscle attached internally on the speculum, just above the **mesopleural apodeme** (map Figs. 9A, 9D).

The mesodiscrimen corresponds internally to a thin, sheet-like endosternal ridge, the **mesodiscrimenal lamella** (mdl Figs. 8B, 9A, 9D). The lamella is a longitudinal, vertical septum, which is very low anteriorly but rises gradually posteriorly to just beneath the base of the mesofurcal arms, where it attaches along the entire anterior margin of the mesofurcal base. The smallest pits at the anterior end of the mesodiscrimen split up in a Y-shaped manner, marking by that the posterior margin of the almost vertical hypocnemium (hum Figs. 5A, 7A). The region surrounding the posterior half of the discrimen is equipped with up to 30 rather long setae.

The posterior margin of the ventral surface of the mesopectus, behind the mesocoxal foramina (mcf Fig. 7A, 8B, 10A), is slightly concave. The posterior margin of the mesopectus and the anterior margin of the metapectal-propodeal complex are both folded and curved inwards, being J-shaped in profile, such that they lock with each other.

##### Biosteres carbonarius

In *B. carbonarius*, the mesepimeral flange is glabrous. The mesepimeral sulcus is widest at its ends and is more of a furrow consisting of irregularly sized pits than a thin and distinct line. In the subalar region, the subalar pit is less distinct, forming more of a natural continuation of the mesepimeral sulcus and blending in with the remainder of the mesopectal sculpturing.

The subalar bridge carries just a few rather long setae. The hypocnemium is densely setose and there are some scattered setae on most of the remaining parts of the mesopectus except for the midlateral region. The setae on the ventral surface and the hypocnemium are much shorter than the other mesopectal setae.

The dorsal end of the mesepimeral sulcus is connected to a lacunose **prespecular sulcus** (prs Fig. 5B), which runs in a bow-like manner along the ventral margin of the subalar bridge until it reaches the anterior margin of the mesopectus, just outside the epicnemium. The prespecular sulcus is shallowest and broadest in the middle and deepest and narrowest anteriorly, Internally, the sulcus correspond to the **prespecular ridge** (ppr Fig. 8B), serving as the site of origin for the anterior mesopleuro-mesofurcal muscle. The very distinct mesopleural scrobe is more than three times as long as the mesepimeral flange is wide where they meet.

The ventral surface of the mesopectus is mostly smooth except for the hypocnemium, which is lightly wrinkled but not delineated by an epicnemial or hypocnemial carina. The mesodiscrimen consists of a row of similar-sized uneven pits and ends posteriorly in a distinctly larger mesofurcal pit. The distance between the two mesocoxal foramina is approximately the same as their diameter.

#### Metanotum

##### Opius dissitus

The **metanotum** (mtn Figs. 5A, 6A, 9A, 10A) is an oblong transverse sclerite with its anterior as well as its lateral margins strengthened. The anterior margin is attached in a hinge-like fashion to the mesopostnotum (mpn Figs. 9B–C, 9E), which is situated behind and underneath the mesoscutum (msc Figs. 5A, 6A). Its lateral and posterior margins are connected to the surrounding metapectal-propodeal complex by membrane situated in an, oblique **metanotal-propodeal fissure** (mpf Fig. 5A). The entire anterior margin is sparsely setose. The **metascutellar disc** (med Figs. 5A, 6A, 10A) is small, slightly elevated, and subrectangular in shape. It is equipped with a small elevated subtriangular crest, which is widest posteriorly and furnished with 5–6 setae dorsally.

The **metascutellar trough** (met Fig. 6A) is subtriangular. The posterior margin of the trough, the **posterior bar of metascutellum** (pba Fig. 6A), is broad and furnished with about ten setae on the apical half. The trough slants distally into a deep, apophysis-marking **metanotal pit** (mnp Fig. 6A) and ends apically in a flexible, spoon-like **metanotal wing process** (mtw Figs. 5A, 6A). On the posterior margin of the metanotum, there is a small **median metanotal notch** (mnn Fig. 6A).

##### Biosteres carbonarius

Instead of carrying a subtriangular crest, the metascutellar disc is equipped with a longitudinal, thick carina. The metascutellar disc is somewhat wrinkled. The entire metanotum is sparsely hairy, except for the metascutellar trough, which is glabrous.

#### Metapectal-propodeal complex

##### Opius dissitus

A faint foveolated - lacunated **metapleural sulcus** (mts Figs. 5A, 6A, 10A) runs from the **metacoxal condyle** (mtc Figs. 7A, 10A) and up to slightly beneath the **propodeal spiracle** (pps Figs. 5A, 6A, 10A), apparently marking the fusion line of the first abdominal tergum, the **propodeum** (ppo Figs. 5A, 6A, 9A, 10A), and the **metapectus** (mpe Figs. 5A, 6A, 7A, 8B, 10A). Anterior to the spiracle, the line is even less obvious. It ends at the laterodorsal margin of the metapectal-propodeal complex, close to the posterolateral corner of the metanotum. The arched **metapleural scrobe** (mtb Fig. 5A) is about two thirds the length of the mesopleural scrobe. It runs from the anterior margin of the metapectus, in height with the propodeal spiracle, and ends in a distinct pit, which corresponds internally to the metapleural apodeme (see below). The scrobe divides the lateral area of the metapectus into an upper anterior part and a lower posterior part. The upper area is superficially sculptured anteriorly and bears about twenty setae. The lower are is smooth with approximately ten setae.

The ventral surface of the metapectus, the **metasubpleuron** (spl Figs. 7A, 8A–B, 9A, 10A, 10E), is almost entirely glabrous, although it becomes increasingly wrinkled posteriorly. Its anterior margin is slightly curved inwards. The lateral margins converge strongly posteriorly and the posterior margin is only about one third the length of the anterior margin, making the metasubpleuron trapezoid-shaped with the posterior “top” missing. Throughout their entire length, the lateral and posterior margins border the large posterior cavity formed by the petiolar and metacoxal foramina.

The entire anterior margin of the metasubpleuron is marked by a transverse **paracoxal sulcus** (pcs Fig. 7A), composed of 30 – 40 small pits in a narrow groove that internally is the attachment for the **paracoxal ridge** (pcr Figs. 8A, 10E), a slanting lamella that connects dorsally to the **metafurcal arm** (far Figs. 8A–B, 10E). The paracoxal ridge separate an anterior narrow area of the metapleuron, from which the anterior metapleuro-metasubalar muscle originates. This area is venterolaterally furnished with a distinct **metabasalar apodeme** (mba Fig. 10E). The lateral end of the metafurcal arm is attached through a muscle to the interior metapleural wall, just above the **metapleural apodeme** (mta Fig. 9A). There is no trace of any **metadiscrimen** or **metadiscrimenal lamella**, but the **metafurcal pit** (mtp Figs. 7A, 10A) is conspicuous, situated close to the center of the metasubpleuron. The metafurca has a lower but thicker furcal base (fub Figs. 8A–B) than the mesofurca.

The anterolateral corners of the metasubpleuron are drawn out into a small cone-like tooth, the **lateral metepisternal projection** (lmp Figs. 5A, 7A, 10A, 10E). The projection is glabrous and comparably small, in size equal to the (lateral) meso**-** and metacoxal condyles (mcc, mtc Figs. 7A, 10A).

Medially and anteriorly, the propodeum is smooth with scattered setae. Its lateral areas are superficially sculptured whilst its posterior third is increasingly wrinkled. Each side of the **propodeal foramen** (pfo Fig. 10A) is equipped with a distinct **propodeal tooth** (ppt Fig. 10A) and beneath it the **propodeal acetabulum** (pac Fig. 10A) fitting in the corresponding petiolar condyle (see below). The propodeal area around and between the acetabula is strengthened, as indicated by its external roughness.

The propodeal and metacoxal foramina are continuous and surrounded by a **metapectal-propodeal rim** (mpr Figs. 7A, 10A). The rim is flat and relatively low above and below the propodeal foramen but it is raised into a posteriorly directed flange around each **metacoxal foramen** (mxf Figs. 7A, 8B, 10A). Above the propodeal foramen, the rim is drawn out into a small dorsolateral projection on each side.

##### Biosteres carbonarius

The metapectal-propodeal rim is more distinctly defined throughout most of its length in this species. The dorsolateral corners, above the propodeal foramen, are smoothly rounded and do not project as in *O. dissitus*.

The propodeum is more heavily sclerotized than in *O. dissitus*. It is sparsely setose and more or less heavily and irregularly wrinkled; the coarseness of the sculpture as well as the pattern seems to differ a lot between different specimens. Quite often there is a distinct longitudinal carina just mesad of the spiracle.

The metapectus is not as heavily sculptured as the propodeum but it is distinctly more sculptured than the metapectus of *O. dissitus*. There is no obvious metapleural scrobe but the metapleural pit is distinct, even though the dark color of this body region makes it difficult to detect except in the SEM. The metasubpleuron is not distinctly set off laterally, unlike *O. dissitus*. The transverse paracoxal sulcus is marked more by a furrow with heavy transverse sculpture than by a row of pits. The metafurcal pit is very large and distinct. The lateral metepisternal projections (lmp Figs. 5A, 7A, 10A, 10E) are much larger; noticeably larger than the meso- and metacoxal condyles.

#### Wings

Both species have wings that are entirely transparent. The dorsal membrane is sparsely setose basally, but more densely setose apically.

#### Fore wing

##### Opius dissitus

**Figure 11:**
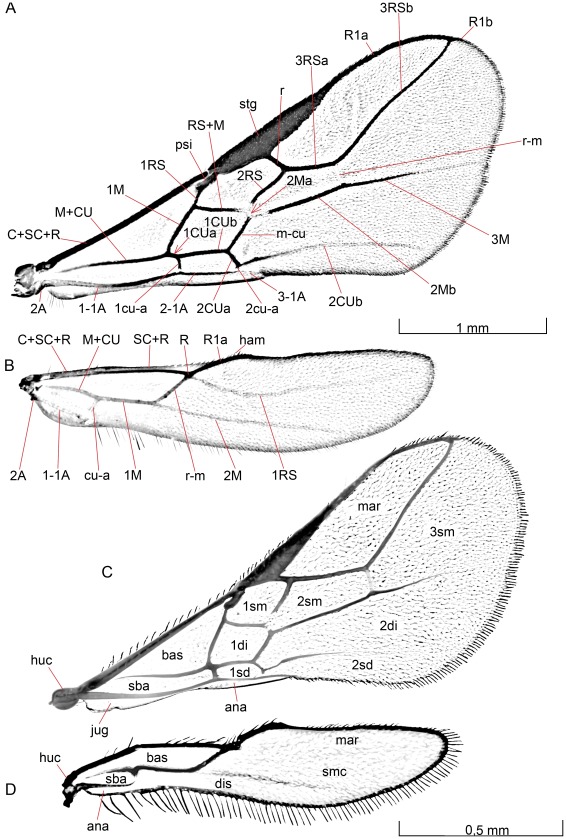
Wings. 12A–B *B. carbonarius*, 12C–D *O. dissitus*.

The entire anterior margin of the **fore wing** (Fig. 11C) (i.e. [C+SC+R]+stg+R1a) is equipped with setae. The setae are comparatively long and rather scattered basally (only about 15 proximal to the pterostigma), shorter and more densely situated apically. At its base, the composite anterior fore wing vein (**C+SC+R**) is covered by the **humeral complex** (huc Fig. 11C). The composite vein successively narrows distally. In dorsal view, it is about twice as thick basally as it is just before the **pterostigma** (stg Fig. 11A). Apically, the composite vein ends in a slight swelling, which fits into an anterior notch in the small **parastigma** (psi Fig. 11A), like a condyle into an acetabulum.

The pterostigma is distinctly wedge-shaped with the proximal third of the posterior margin slanting rather steeply from the anterior margin, while the distal two thirds slant less steeply back towards the anterior margin. The pterostigma is about one fourth as long as the anterior margin of the fore wing. The distance between the wing base and the base of the pterostigma is slightly more than 1.5 times the length of the pterostigma itself. The parastigma is separated distally from the pterostigma by a narrow membranous strip, whilst it is firmly joined to the vein **1RS** basally (Fig. 11 A). Apically, the pterostigma more or less gradually transitions into the vein R.

Vein **M+CU** (Fig. 11A) is rather slender, especially proximally, and becomes increasingly sclerotized apically up to the point where the two merged veins separate from each other. After the separation, vein **1M** turns obliquely anteriorly to meet 1RS and fuse with the latter to become **RS+M**. RS+M is mainly spectral; it leaves 1RS and 1M in a perpendicular angle and fades out in a bulla apically. The small vein 1RS is just about one third the length of the parastigma, whilst veins 1M and RS+M are subequal in length.

RS+M splits up in the bulla into an anterior, obliquely angled second abscissa of RS, i.e. **2RS**, and a posterior apical abscissa of M, i.e. **2M**. 2RS is equal in length to 1M and RS+M. RS meets a short, posteroapically directed cross vein **r** close to the pterostigma. The cross vein r is much shorter than the width of the pterostigma. After joining r, RS continues apically in the shape of **3RS** as a tubular vein all the way out to the wing margin, where it meets R, dividing the latter into a long, basal **R1a** and a very short, apical **R1b**.

**2M** runs shortly in the bulla before it meets the cross vein **1m-cu** that separates the longitudinal vein into a short, spectral, posteriorly directed **2Ma** and a longer, apically directed **2Mb**. The latter is tubular in all its length. Some distance out it is connected to the more apical longitudinal vein RS by the very faint spectral cross vein r-m, separating **3RS** into a proximal **3RSa**, about half as long as the pterostigma, and a long distal **3RSb**. 3RSa is about 1.5 times as long as 2RS. The M vein extends apically as the abscissa **3M** that fades out and does not reach the wing margin even as a spectral vein. The M vein is slightly angled in height with the cross vein 1m-cu but 3M forms a direct prolongation of 2M.

After the split of **M+CU**, the latter continues in the same direction as the fused veins even though 1cu-a is attached to it just distal to the separation. **1CU** is similar in length to the anterior parallel RS+M and but slightly shorter than the proximal 1M, but slightly longer than 1m-cu. The cross vein **2cu-a** divides the distal part of CU into a short posteroapically directed **2CUa** and an apically extending **2Cub** that fades out and disappears well before reaching the wing margin. 2cu-a is just a short stub, leaving a gap between its posterior end and the anal-vein.

The anal vein is smoothly sinuate and runs close to the hind margin of the fore wing, separated by the cross veins described above into a long proximal **1–1A** abscissa, a second **2–1A** abscissa, similar in length to the parallel **1CU**, and a third, small **3–1A**, which almost immediately fades out distally.

There is no **costal cell** anterior to vein C+SC+R. The veins define a closed, rather big and triangular **basal cell** (bas); a closed, long and narrow **sub-basal cell** (sba); a closed, askew rectangular **1st submarginal cell** (1sm); a closed, more quadratic **1st discal cell** (1di); a quite small pentagonal **1st subdiscal cell** (1sd); a closed, big **marginal cell** (mar); a closed pentagonal **2nd submarginal cell** (2sm); a long and widely open **2nd discal cell** (2di); a long and open **2nd subdiscal cell** (2sd), and finally a big, apically widely open **3rd submarginal cell** (3sm) (cf. Fig. 11C). A basal **jugal lobe** (jug Fig. 11C) and an open **anal cell** (ana) are situated at the posterior margin of the fore wing, both anteriorly delineated by the anal vein. The somewhat drawn out, bowl-shaped jugal lobe is ventrally sparsely setose. It reaches from the wing-base out to almost the middle of the abscissa 1–1A. The anal cell is open posteriorly and is extremely narrow basally, up to about two thirds the length of 1–1A, after which it slowly widens into a banner-shaped area.

##### Biosteres carbonarius

The anterior edge of the fore wing (Fig. 11A) bears numerous setae. The pterostigma (stg Fig. 11A) is not wedge-shaped like in *O. dissitus*; it is long and quite narrow with a smoothly bent posterior margin. The pterostigma is shorter than one fourth of the length of the anterior margin of the fore wing, and the distance between the wing base and the base of the pterostigma is slightly less than 1.5 times the length of the pterostigma itself.

The small vein 1RS is slightly longer than the parastigma (psi Fig. 11A). 2RS, 1M and RS+M are all equal in length. Cross vein r is equal in length to the width of the parastigma. Vein R1a is slightly shorter than the pterostigma. 2RS and 3RSa are subequal in length; 3RSb is about three times as long as 3RSa and the latter is only one third of the length of the pterostigma.

**2M** runs shortly in the bulla before it meets the cross vein **1m-cu** that separates the longitudinal vein into a short, spectral, posteriorly directed **2Ma** and a longer, apically directed **2Mb**. The latter is initially spectral, even though most of its length is tubular.

Cross vein 1cu-a is more distinctly postfurcal, making 1CUa longer relative to 1CUb than in *O. dissitus*. 1CU(a+b) is similar in length to the three veins RS+M, 1M and cross vein 1m-cu. Anal vein abscissa 2-1A is 10% longer than 1CUb.

#### Hind wing

##### Opius dissitus

A faint **humeral complex** (huc Figs. 11D) covers the base of the composite vein **C+SC+R** (Fig. 11B) of the **hind wing** (Fig. 11D). At the apical end of the combined vein, it meets cross-vein **r-m**. The single vein **R** issues from this point but it branches almost immediately into **R1a** and **1RS**, the latter of which is completely missing except for an indication of its base. R1a runs at an angle towards the anterior margin. After reaching the anterior margin, it bends distally and runs along the margin for a short distance before quickly fading out into a marginal nebulous vein or just a darkly colored anterior wing margin. On R1a, where the latter joins the anterior wing margin, there are three distal **hamuli** (ham Fig. 11B) situated among some straight, long setae. A series of straight, anteriorly directed setae are situated on the basal part of C+SC+R. The distal abscissa of M, **2M**, is a vague spectral vein, which almost reaches the distal margin of the wing. Only the **basal** (bas) and **sub-basal** (sba) cells are closed.

##### Biosteres carbonarius

The composite vein C+SC+R is equipped with about 15 rather long setae basally (Fig. 11B). 1RS is present, although it is spectral, very vague and does not reach the wing margin. Distally, it bends slightly anteriorly. The spectral 2M is straight and slightly more marked than in *O. dissitus*.

#### Legs

##### Opius dissitus

The **legs** are pale dirt-yellowish in color and relatively long and slender. The middle legs are about 10% longer than the fore legs and the hind legs between 20 and 25% longer than the middle legs.

##### Biosteres carbonarius

The legs are yellow but the basal half of the meta coxa is black. The fore and middle legs are of equal size, the hind legs about 25% longer.

#### Fore leg

##### Opius dissitus

**Figure 12:**
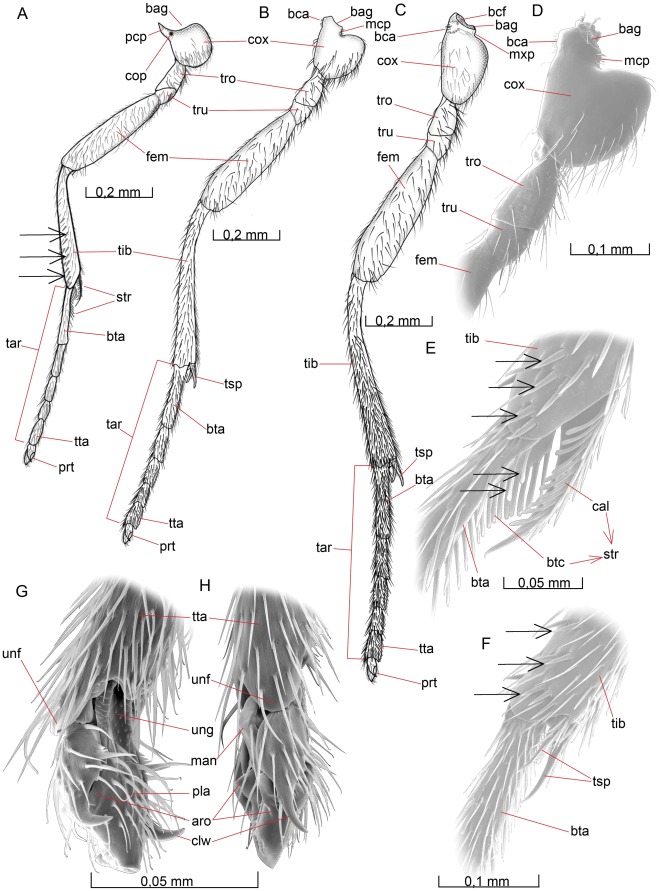
Details of legs. 10A-H *O. dissitus*. (A) Fore leg. Posterior, outer view. (B) Mid leg. Anterior, outer view. (C) Hind leg. Anterior, outer view. (D) Proximal segments of mid leg. Lateral, outer view. (E) Details of fore leg. Posterior, outer view. (F) Details of mid leg. Anterior, outer view. (G) Fore leg tarsus, posteroventral view. (H) Fore leg tarsus, posterodorsal view.

The **procoxa** (cox Figs. 5A, 12A) differs noticeably in morphology from the meso- and metacoxa (cox Figs. 5A, 10E, 12B–D). The **probasicoxal foramen** (bcf Figs. 12C) and its encircling **basicoxal girdle** (bag Figs. 12A) are drawn out laterally, on its anterior side, into a distinct process. This semi-flat **procoxal process** (pcp Fig. 12A) is distally smoothly bent and its length is equal to its basal width. The process is directed anterolaterally and fits into the space between the strengthened ventrolateral posterior edge of the propleuron (ppl Figs. 5A, 7A, 10B) and the posteroventral border of the pronotum (pno Figs. 5A, 6A, 7A, 8A–B, 9A). Its apex serves as the articulation point between the fore leg and the propleuron. A distinct and deep **procoxal pit** (cop Fig. 12A). is situated basally on the posterior surface of the procoxal process. It is the external evidence of an internal apophysis. A patch of less than ten short, stout mechano-sensory setae are located lateroventrally on the distal tip of the procoxal process and another patch consisting of about 8 – 10 similar mechano-sensory setae are situated on the body of the procoxa, close to the procoxal process but mesad to the basal girdle.

The body of the procoxa (coxal process excluded) is almost circular in anterior view but more semicircular in dorsal view. The width exceeds the height with about one fifth. The proximal half and the distal section of the procoxa are evenly covered with about 40 relatively long setae each, while the median portion is glabrous.

The cone-shaped **protrochanter** (tro Fig. 12A) of the fore leg is twice as wide distally as it is proximally. It has a foot-like, broader base. The distal margin slopes rather steeply, so that its ventral side is shorter than its dorsal ditto. The posterior surface of the protrochanter is sparsely furnished with about 25 – 30 setae whilst the anterior surface is more or less glabrous.

The **profemur** (fem Figs. 12A) has a sub-basal constriction marked by a narrow line that sets off the proximal **protrochantellus** (tru Fig. 12A) from the remainder of the profemur. The latter is about half as long as the protrochanter. Like the latter, it is setose posteriorly but almost glabrous anteriorly.

The remainder of the profemur is subcylindrical and about four times as long as its maximal width. It is moderately setose, somewhat more sparsely on the anterior surface. Its distal edge is evenly arched dorsally, while it is deeply notched ventrally to allow increased mobility of the protibia. On each side in the distal margin, there is a small hollow for the corresponding protibial condyle.

The **protibia** (tib Figs. 12A, 12E) is distinctly curved basally. It is almost identical in length with the profemur (the protrochantellus included). It gradually becomes thicker towards its distal end, where it is almost twice its proximal width. The entire protibial surface is rather sparsely setose. In the distal half of the protibia, on the posterior and outer surfaces, there are several irregular rows with a total of about 20 somewhat shorter but considerably thicker protibial spine-like setae (arrows Fig. 12A and dorsal arrows in 12E).

The distal edge of the protibia is deeply notched for increased movability of the protarsus. The **calcar** (cal Fig. 12E), which is the protibial part of the **strigil** (str Figs. 12A, 12E), is relatively large and distinctly curved. Its length is about twice the width of the protibial apex. It has the shape of a smoothly bent, long and narrow spur. In cross section it is triangular, with the apex pointing inwards, towards the protarsus, forming a sharp scraper-like edge. The broad outer surface is equipped with two rows of flat hairs reminiscent of a toothcomb. Each row consists of about 8 – 10, hairs of equal length, perpendicular to the longitudinal axis of the calcar.

The **protarsus** (tar Fig. 12A) has five tarsomeres. It is more densely setose than the remainder of the fore leg. The **tarsomeres** are sub-cylindrical. The **probasitarsus** (bta Figs. 12A, 12E) constitutes approximately one third of the protarsus. The **basitarsal comb** (btc Fig. 12E) forms the tarsal part of the strigil. It consists of a distinct crest of uniform thickness, which decreases in height distally. The crest is furnished with 15 – 20 flat hairs similar to the ones on the calcar (cal Fig. 12E). The hairs of the strigilar comb differ somewhat in size. They are shortest in the middle and distally become more and more similar to the normal setae so that the distal end of the strigilar comb is hard to define even though the crest ends before the middle of the basitarsus. On the posterior side of the probasitarsal comb, close to its proximal end, there are two a8nt, slightly paddle-shaped setae (ventral arrows in Fig. 12E).

The second tarsomere is slightly longer than half the length of the probasitarsus (∼60%). The distal three tarsomeres are approximately equal in length, although tarsomere 4 is somewhat shorter. Together they are somewhat longer than the probasitarsus (∼120%) or about twice as long as tarsomere 2. The distal edge of the fifth or last tarsomere, the **protelotarsus** (tta Fig. 12A), is provided with two opposed, flap-like processes, one on its anterior and one on its posterior side. These are the two **unguifers** (unf Figs. 12G–H), with which the pretarsal claws articulate.

The **pretarsus** (prt Fig. 12A) of the protarsus is supported ventrally by the **unguitractor** (ung Fig. 12G), which is not clearly separated from the more apically situated **planta** (pla Fig. 12G). The ventral surface of the unguitractor is sparsely covered medially with about 15 minute, knob-like projections. Its lateral edges are imbricate. The unguitractor is partly inserted into the end of the protelotarsus and its proximal end is attached through an apodeme to the muscle that is responsible for the maneuvering and movement of the pretarsus of the protarsus. The planta is provided with about 20 long setae, the distal ones slightly shorter.

Each **tarsal** **claw** (clw Figs. 12G–H) consists of a disc-shaped, basal part and an equally long, hook-like distal part. The disc-shaped part is sparsely setose dorsally, laterally and ventrally. The ventral margin of the disc is also supplied with four broader, distinctly fluted setae. The most apical of these is almost the same length as the other setae on the claw; the three more basal ones are much shorter, cone-like and gradually become shorter towards the base of the claw. The distal hook-like part of the claw is supplied mid-dorsally with a seta that almost reaches the tip of the claw.

The **arolium** (aro Figs. 12G–H) is hollow, apparently membranous and artificially wrinkled in the SEMs. Basally to the arolium there is a distinct **manubrium** (man Fig. 12H) equipped with a few rather long setae.

##### Biosteres carbonarius

Virtually identical with *O. dissitus*.

#### Mid leg

##### Opius dissitus

The **mid leg** (Fig. 12B) is similar to the fore leg in sculpture and pubescence. The **mesocoxa** (cox Figs. 5A, 12B, 12D) is distinctly different from the procoxa, being heart-shaped in posterior view, and more distinctly compressed anteroposteriorly. The almost circular **basicoxal girdle** (bag Figs. 12B, 12D) is smaller and situated entirely on a large dorsolateral projection. Two small patches of short and stout mechanosensory setae are situated on the dorsolateral projection: one hair patch is attached laterally, adjacent to the **basicoxal acetabulum** (bca Figs. 12B, 12D), and one mesal hair patch is situated on the inner side of the **mesocoxal process** (mcp Fig. 12B, 12D). At the distal end of the latter, the basicoxal acetabulum articulates with the **mesocoxal condyle** (mcc Figs. 7A, 10A). The opposite side (the mesal side) of the coxa moves loosely against the strengthened mesal rim of the mesocoxal foramen; there is no second, median mesocoxal articulation.

The **mesotrochanter** (tro Figs. 12B, 12D) and **mesofemur** (fem Figs. 12B, 12D) are similar in structure to those of the fore leg. The **mesotibia** (tib Figs. 12B, 12F) lacks a strigil; instead, there are two ordinary **tibial spurs** (tsp Figs. 12B, 12F) attached to the distal end of the mesotibia. The length of these spurs is about the same as the distal width of the mesotibia. Both spurs (Fig. 12F) are furnished with approximately 8 – 10 setae, each seta about half the length of the spur.

The **mesobasitarsus** (bta Figs. 12B, 12F) of the midleg lacks a basitarsal comb. Except for this, the **mesotarsus** (tar Fig. 12B) and **pretarsus** (prt Fig. 12B) of mesotarsus are similar to the serially homologous parts of the fore leg.

##### Biosteres carbonarius

Almost identical with *O. dissitus*.

#### Hind leg

The hind leg (Fig. 12C) is similar to the midleg in the relative length of the different parts, including the apical **metatibial spurs** (tsp Fig. 12C). The sculpture is also similar to the midleg but the pubescence is much denser in the distal half of the hind leg, the density increasing distinctly from slightly before the middle of the **metatibia** (tib Fig. 12C).

The **metacoxa** (cox Figs. 5A, 10E, 12C) is egg-shaped in posterior view, about 1.5 times as high as wide, and slightly compressed anteroposteriorly. The **metabasicoxal girdle** (bag Figs. 12C) is fairly large and situated on a much less prominent projection than the corresponding part of the midleg.

The **metatrochanter** (tro Figs. 12C), **metafemur** (fem Fig. 12C), **metatarsus** (tar Figs. 12C) and **pretarsus** (prt Fig. 12C) are all similar to the homologous parts of the midleg except for the pubescence.

##### Biosteres carbonarius

Almost identical with *O. dissitus.*

#### Metasoma

##### Opius dissitus

The **metasoma** is sparsely setose. Its coloration varies from almost entirely pale yellow in recently hatched specimens to a pale yellow anterior and an almost black posterior.

##### Biosteres carbonarius

The metasoma is dark brown to black, with slightly less dark **laterotergites**. Each metasomal tergum, from T3 to T8, bears a transverse row of setae, which ends laterally in a scattered patch of setae below each gastral spiracle. The metasomal sterna are more setose than the metasomal terga, and they are unusually heavily sclerotized for ichneumonoids.

#### Petiole

##### Opius dissitus

**Figure 13:**
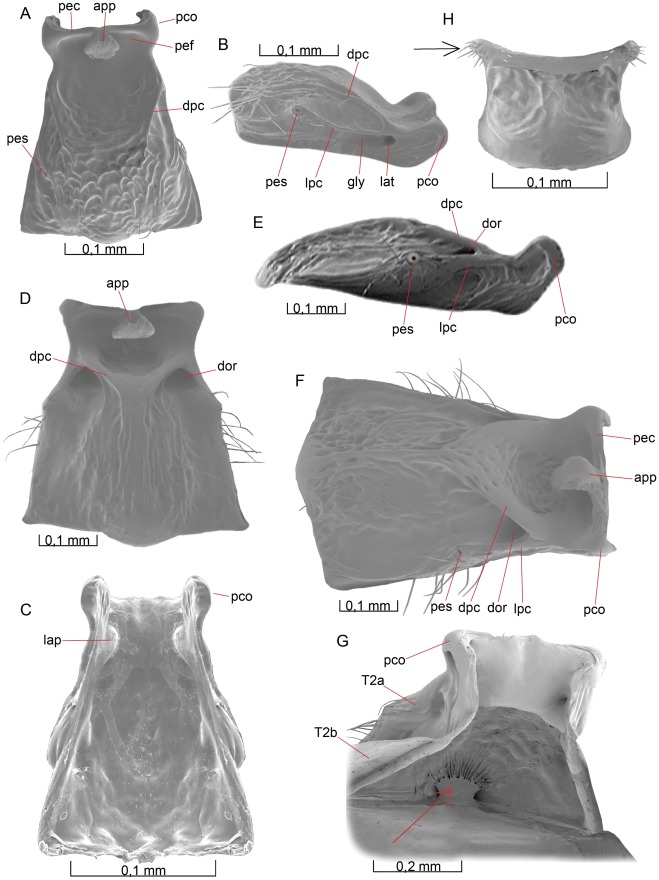
Details of petiole. 13A–C *O. dissitus*, 13D–G *B. carbonarius*. (A) Petiole, dorsal view. (B) Petiole, dorsolateral view. (C) Petiole, ventral internal view. (D) Petiole, dorsal view. (E) Petiole, lateral view. (F) Petiole, dorsolateral view. (G) Petiole, anteroventral, internal view.

**Figure 14:**
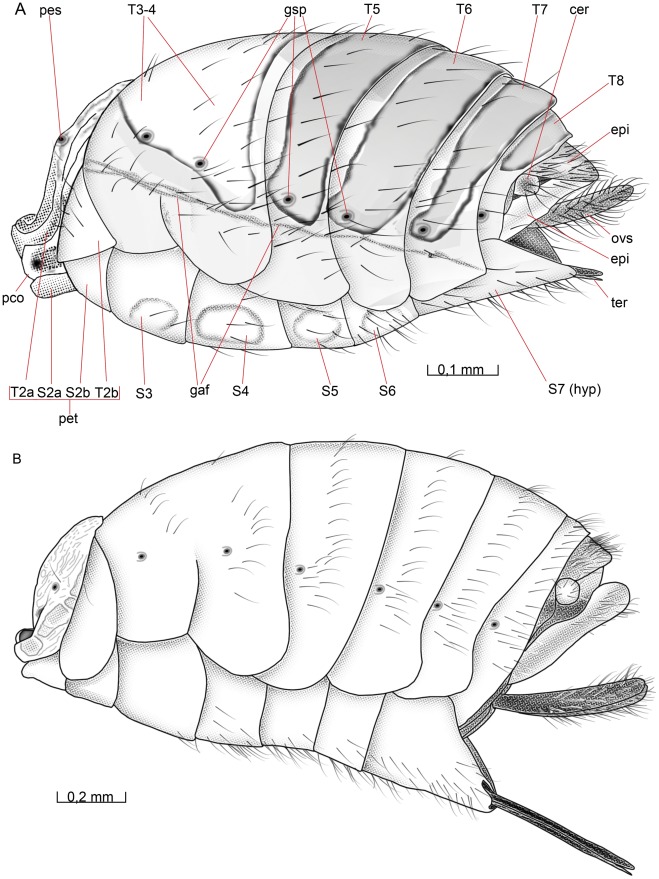
Metasoma, lateral view. 14A *O. dissitus*. 14B *B. carbonarius*.

**Figure 15:**
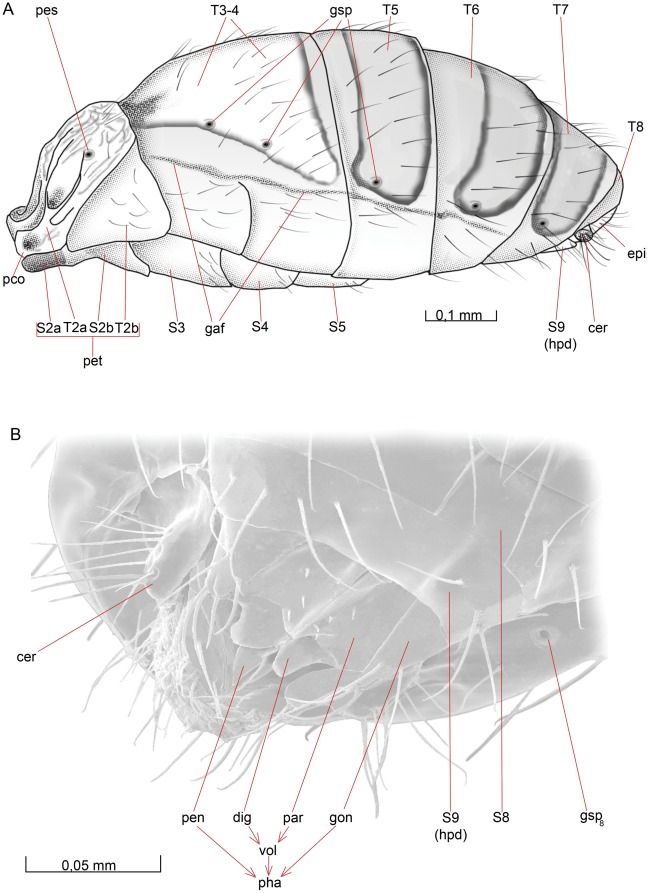
Male metasoma of *O. dissitus.* (A) Lateral view. (B) Distal segments in ventral view.

The **petiole** (pet Figs. 13A–C, 14A, 15A) consists of five sclerites. The tergum of the petiole is divided into a firmly sclerotized **mediotergite** (T2a Figs. 13A–G, 14A, 15A) and two **laterotergites** (T2b Figs. 13G, 14A, 15A), one attached to each side of the mediotergite. The sternum of the petiole is separated into a sclerotized **anterior area of the second abdominal sternite** (S2a Figs. 7A, 13H, 14A, 15A) and a soft, membranous **posterior area of the second abdominal sternum** (S2b Figs. 14A, 15A).

The mediotergite of the petiole is heavily sclerotized and its entire ventral margin is strengthened into a swollen rim. It is yellow but may have its anterior part, up to one third of the sclerite, black. Its posterior margin is about 1.5 times as wide as its anterior margin. A slight constriction separates an anterior **petiolar collar** (pec Figs. 13A, 13F) from the rest of the mediotergite. The petiolar collar is equipped with two shallow but distinct, well separated **petiolar foveae** (pef Fig. 13A). Medially, between the foveae, there is a conspicuous peg-like process, the **anterior petiolar process** (app Figs. 13A, 13D, 13F), which fits into the small dorsal section of the propodeal foramen (pfo Fig. 10A) above the propodeal teeth (ppt Fig. 10A). Laterally of each petiolar fovea, the collar forms a swelling, on the anterior side of which the **petiolar condyle** (pco Figs. 13A–C, 13E–G, 14A, 15A) is situated.

The **petiolar spiracle** (pes Figs. 13A–B, 13E–F, 14A, 15A) is situated close to the lateral margin of the mediotergite, slightly behind the middle of the part posterior to the petiolar collar. The petiole is equipped on each side with a distinct **dorsal petiolar carina** (dpc Figs. 13A–B), which originates from the dorsal rim of the petiolar condyle. The two carinae converge slightly at first but then run in parallel, well separated, before fading out in level with the petiolar spiracle. Another carina, the **lateral petiolar carina** (lpc Figs. 13B, 13E–F), runs anteriorly from the spiracle, meeting the dorsal petiolar carina close to the petiolar collar. The **glymma** (gly Figs. 13B) is situated immediately beneath the dorsolateral carina. The glymma fades out posteriorly but becomes deeper and deeper anteriorly, where it ends in a deep **laterope** (lat Figs. 13B), internally visible as a **lateropal apophysis** (lap Fig. 13C). There is no dorsope.

The posterior margin of the median tergite, and the adjacent region, are distinctly raised to form a prominent rounded median elevation. The elevation occupies about one fifth of the total posterior width of the median tergite. The posterolateral corners of the median tergite, lateral to the median elevation, are flat and shaped like dog ears in anterior view.

The median tergite is nude except for a patch of three to four long setae on each side of the median posterior elevation. The anterior half of the sclerite is largely smooth, whilst the posterior half is irregularly costate.

The weakly sclerotized **laterotergite** (T2b Figs. 13G, 14A, 15A) of the petiole is of the same pale dirt-yellowish color as the legs. It is shaped like an equilateral triangle and furnished with six to eight setae, which are evenly scattered over the sclerite except for the anterior third.

The **anterior area of the second abdominal sternum** (S2a Figs. 7A, 14A, 15A) is firmly sclerotized, brown-yellowish and coriaceous in sculpture. It is slightly longer than the petiolar collar and its anterior edge is ventrally in the shape of a strengthened rim, which in turn is as thickest laterally where it houses a patch (black arrow Fig. 13H) of about 8 short setae. Anteriorly it abuts, but is not fused to, the mediotergite of the petiole. Posteriorly, it is fairly widely separated from the mediotergite by membrane. The **posterior area of the second sternum** (S2b Figs. 14A, 15A) is pale white and almost entirely membranous.

##### Biosteres carbonarius

The petiole (Figs. 13D–F) of *B. carbonarius* is entirely black. Its posterior margin is about 1.5 times as wide as its anterior margin. The petiolar foveae (pef Fig. 13A) are confluent, forming one big central depression on the petiolar collar (pec Figs. 13A, 13F). The petiolar spiracle (pes Figs. 13F) is situated slightly in front of the middle of the part of the mediotergite posterior to the petiolar collar. The dorsal petiolar carinae (dpc Figs. 13D–F) are prominent. They converge strongly to form a median longitudinal crest at about the height of the petiolar spiracle. This median crest posteriorly reaches the posterior petiolar elevation. Lateral to the dorsal carina, there is a large and distinct **dorsope** (dor Figs. 13D–F). The glymma (gly Figs. 13B) is shallow and wide; it does not end in a laterope. The mediotergite is equipped with some scattered setae posteriorly and about 8 – 10 rather long setae laterally, just beneath the spiracle.

The laterotergite is almost glabrous, dark brown and more elongate than in *O. dissitus*. There may be some setae close to the dorsal rim of the laterotergite but otherwise it lacks pubescence.

Internally, the posterior part of the mediotergite serves as the attachment point for a structure that is apparently a fan-shaped muscle, which inserts medially on the anterior margin of the third abdominal tergum (red arrow Fig. 13G). We were not able to determine whether a similar structure occurs in *O. dissitus*.

The second abdominal sternite (S2a Fig. 13H) is small, black and heavily sclerotized. The posterior area of the second sternum has a membranous whitish anterior half and a more sclerotized brownish posterior half.

#### Female Gaster

##### Opius dissitus

The **abdominal terga** T3 – T8 are all equipped with distinct, open and apparently functional **gastral spiracles** (gsp Figs. 14A, 15A–B). The terga T3 – T7 are also rather distinctly divided into a mediotergite and two laterotergites by the **gastral fold** (gaf Figs. 14A, 15A) running from slightly distad the dorsal corner of the lateral petiolar tergite and down to the posteroventral corner of T7 on each side of the body. All the gastral spiracles lie above the gastral fold.

**Abdominal terga 3 and 4** (T3–4 Fig. 14A) are firmly fused into a single **syntergum** but the laterotergites are still separated by a lateral slit, which ends just beneath the gastral fold, pointing towards a line between the two spiracles of the syntergum. The ventral edge of the laterotergite of T3 is relatively straight, while the laterotergite of T4 projects ventrally as a rounded lobe. The posterior edge of the syntergum is about twice the length of its anterior edge, resulting in a marked increase in the metasomal width in these two segments.

More than half the area of the syntergum is occupied by a sub-triangular area defined by internal ridges. This area is indicated in Fig. 14A but it is hard to identify on dry specimens and impossible to see on SEM micrographs. On living animals, or on specimens stored in alcohol, it is visible as an opaque, bright yellow, triangular area surrounded by a dark rim. Outside this rim, the syntergum is semi-transparent and of a more light-brown color. Anteriorly, on each side of the posterior petiolar elevation, there is an oblique, posteriorly converging, rather broad but short and shallow furrow on the syntergum.

The syntergum is equipped with four more or less row-like lines of setae across the entire width of the sclerite. The two posterior rows (upon the primitive T4) are more evenly linear than the two anterior rows (on the primitive T3). The two anterior rows consist of about 20±4 setae, evenly distributed between the mediotergite and the two laterotergites. The two posterior rows are composed of about 30±5 setae, of which two thirds are situated on the mediotergite and the rest on the laterotergites.

**Abdominal terga 5 and 6** (T5, T6 Fig. 14A) are rather similar to each other in shape and sculpture, even though T5 is slightly bigger than T6. The length of the anterior and posterior margins of T5 and T6 are almost equal and the laterotergites of T5 and T6 are both similar in shape to the laterotergite of T4.

The anterior margin of **abdominal tergum 7** (T7 Fig. 14A) is markedly longer than the posterior margin. The laterotergite of T7 differs in shape from the preceding laterotergites in that it is smaller and the posteroventral corner is drawn out to receive the posterior end of the gastral fold.

Abdominal terga 5 – 7 are each furnished with 30 setae arranged in two transverse rows, T5 always with some additional setae relative to T6 and T7. Like the syntergum, each of the mediotergites of T5 – T7 are equipped with a large endodermal thickening. These thickenings are darker than the corresponding thickening of the syntergum, so that the posterior part of the gaster becomes much darker than the anterior part, almost black.

T8 is much smaller and more narrow than T5 – T7 and the endodermal thickening is only visible on the dorsal third of the tergite. While the spiracles on T3 – T7 are situated inside the endodermal thickening of each sclerite, the spiracles on T8 are clearly situated outside.

**Figure 16:**
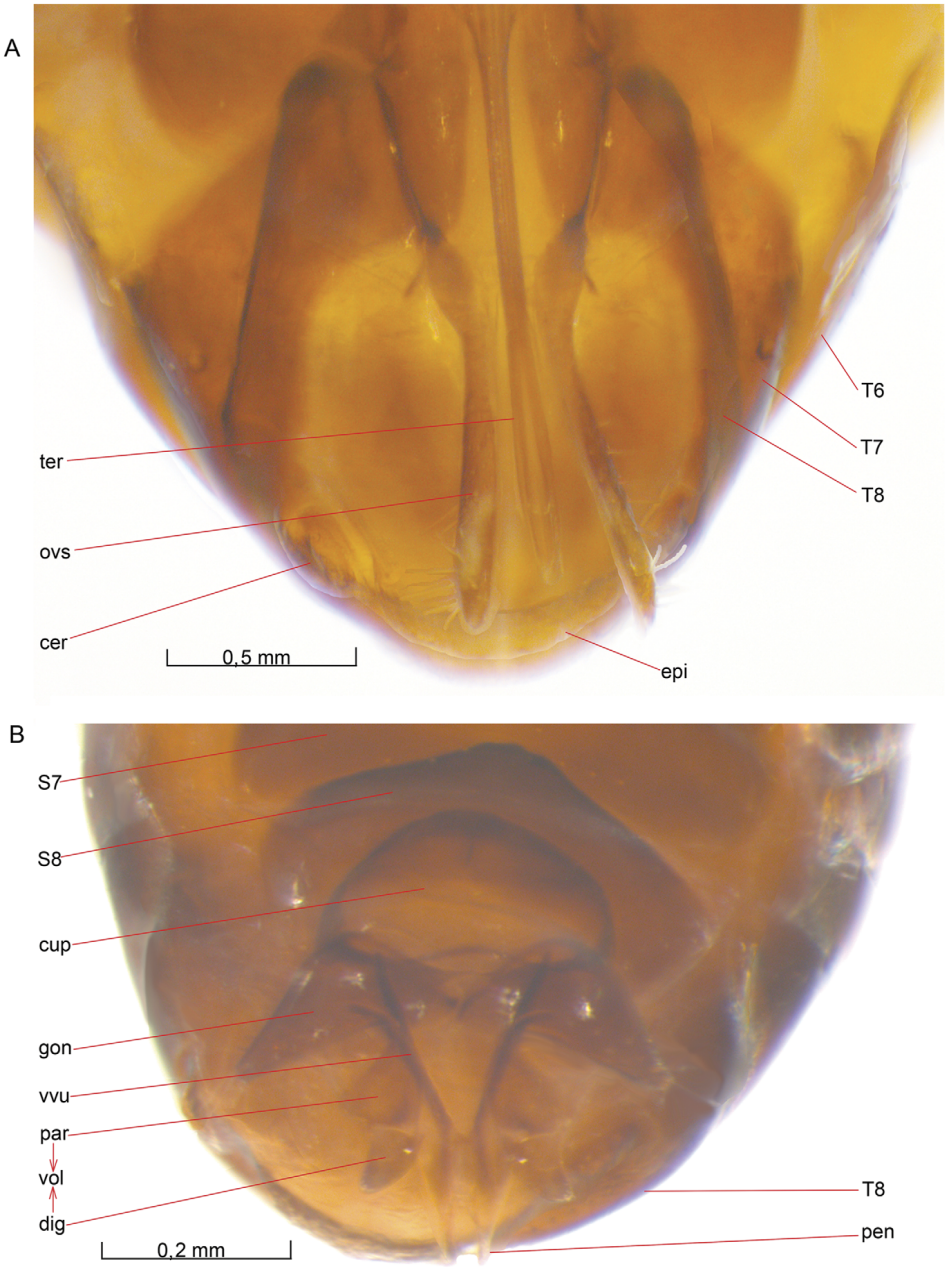
External genitalia of *B. carbonarius*. Ventral view, posterior down. (A) Female. (B) Male.

**Figure 17:**
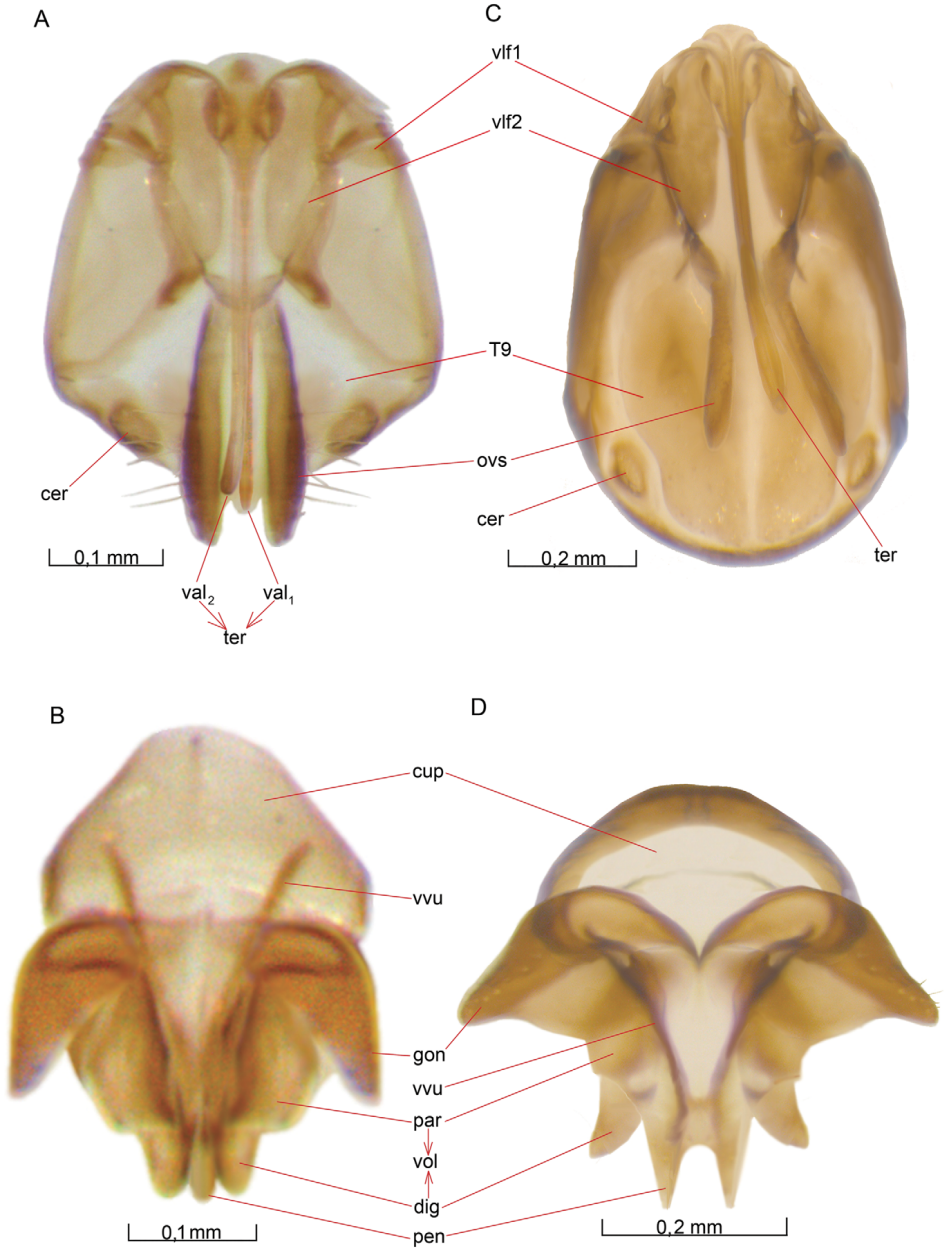
Parts of genitalia. Ventral view, posterior down. 17A–B *O. dissitus,* 17C–D *B. carbonarius* (A) Female. (B) Male. (C) Female. (D) Male.

The **epipygium** (epi Figs. 14A, 15A, 16A) projects slightly posteriorly in its dorsal part but continues in the form of a narrower sclerotized band laterally. Posteriorly and ventrally, the tergum is connected with extensive membranous regions. On each side, the epipygium bears the **cercus** (cer Figs. 14A, 17A) in a small notch. The cercus is shaped like a disc and is equipped with approximately eight setae.

**Abdominal sterna 3 and 4** (S3, S4 Fig. 14A), corresponding to the syntergum, are free sclerites but only lightly sclerotized and largely opaque. Abdominal sternum 3 has 8 – 10 (seldom up to 12) setae in two uneven transverse posterior rows, whilst S4 has a few more setae arranged in a similar pattern. Both sterna have small dorsolateral apodemes (not illustrated) projecting anteriorly.

**Abdominal sterna 5 and 6** (S5, S6 Fig. 14A) are morphologically very similar to S3 and S4 but they are furnished with more setae – up to about 20 – that are not arranged in strict rows but spread more evenly.

**Abdominal sternum 7** or the **hypopygium** (S7, hyp Fig. 14A) is keel-shaped in profile and subtriangular in lateral view; its apex reaches the end of the metasoma. A narrow strip along the outer margin of the hypopygium is transparent and semi-sclerotized but the rest of the hypopygium is strongly sclerotized, especially along the midventral line. The anterior margin is rather concave. Anterolaterally, on each side, the hypopygium is equipped with a small apodeme similar to the ones on S3–S6. In dorsal view, the apex of the hypopygium is smoothly rounded. The hypopygium is sparsely setose laterally, more densely setose ventrally.

Laterally, on the internal surface of each of the sterna S3 – S7, there is a low, ring-like apodeme on each side. The diameter of each ring is only slightly smaller than the anterior-posterior length of the corresponding sternum. The ring-like apodemes are not visible in dried specimens.

##### Biosteres carbonarius

There is no evident gastral fold in *B. carbonarius*, but in KOH-bleached specimens, one can distinguish a line between a thicker median area and a thinner, more easily bleached, lateral area of each tergum. All the gastral spiracles (gsp Figs. 14A, 15A–B) lie above this line. The internal reinforcement of the terga seen in *O. dissitus* is also evident in bleached specimens of *B. carbonarius*. Instead of two irregular transverse rows of setae, each tergum is equipped with one regular transverse row of setae. The sterna are more setose than in *O. dissitus*. Particularly the anterior sterna are unusually well sclerotized for a member of the Ichneumonoidea.

#### Male Gaster

##### Opius dissitus

The male gaster is similar to the female gaster, especially the anterior half. **Abdominal terga 3 – 7** (T3 – T7 Fig. 15A) are similar in shape but have 20% – 30% fewer setae than those of the female. **Abdominal terga 8** (T8 Fig. 15A), the epipygium (epi Fig. 15A) and the **cercus** (cer Figs. 15A–B) are similar to those of the female.

**Abdominal sterna 3 – 6** (S3 – S5 Fig. 15A; S6 not illustrated) are similar to those of the female. **Abdominal sternum 7** (not illustrated) is similar in structure to the immediately preceding sterna but is furnished with fewer setae. **Abdominal sternum 8** (S8 Fig. 15B) is distinctly narrower than sternum 7 but otherwise similar in structure. The ring-shaped lateral apodemes extend from S3 to S8. **Abdominal sternum 9** or the **hypandrium** (S9, hpd Figs. 15A, 15B) is shaped like a boomerang with the posterior margin being slightly concave, and the anterior margin projecting anteriorly inside S8. The sterna become increasingly sclerotized backwards, from S3 to S9. The abdominal sterna 8 and 9 are equipped with only 8 – 10 setae, about half the number of S5 and S6.

##### Biosteres carbonarius

The male gaster differs from that of *O. dissitus* in a way similar to the differences for the female gaster. The hypandrium is distinctly punctate anteromedially; its posterior margin is straight rather than concave.

#### Female External Genitalia

##### Opius dissitus

The ovipositor apparatus is composed of abdominal tergum 9 (T9 Figs. 17A, 17C), the first and second valvifers (vlf1, vlf2 Figs. 17A, 17C), the **terebra** (ter Figs. 14A, 16A, 17A) and the **ovipositor sheaths** (ovs Figs. 14A, 16A, 17A, 17C). The terebra is of the same dark-yellowish coloration as the last two or three abdominal terga, while the ovipositor sheaths are almost black.

The terebra consists of the **first** **and** **second valvulae** (val1 and val2 Fig. 17A). The latter are joined throughout their entire length and the **first valvulae** (val1 Fig. 17A) are attached underneath the second valvulae.

The terebra is typically hidden in-between the two gutter-shaped ovipositor sheaths, which reach slightly past the posterior edge of abdominal epipygium (epi Fig. 14A). Each ovipositor sheath is equipped with around 25 long setae on its outer surface. In dorso-ventral view, the sheaths expand gradually from their base towards the apex, reaching almost three times their proximal width at about four fifths of their length. After this point, they narrow rapidly into a smoothly rounded apex.

##### Biosteres carbonarius

The female external genitalia of *B. carbonarius* are very similar to those of *O. dissitus* except that they extend a bit longer past the tip of the metasoma.

#### Male External Genitalia

##### Opius dissitus

The external male genitalia form a highly flexible complex of sclerites, membranes and muscles. In ventral view, the **cupula** (cup Fig. 17B) has the shape of an octagon cut in half. The cut margin is directed posteriorly and is distinctly emarginate. The length of the cupula is about two-thirds its width. The median, transverse section of the anterior margin is about one third the width.

The firmly sclerotized and cone-shaped **gonoforceps** (gon Figs. 15B, 17B) has a very small, apical incision. Subapically, it is furnished with about five rather long and stout setae. The less sclerotized and more rectangular **parossiculus** (par Figs. 15B, 17B) is about half the size of the gonoforceps, whilst the slightly more sclerotized, cone-shaped **digitus** (dig Figs. 15B, 17B) is glabrous and less than one third the size of the gonoforceps. The parossiculus and the digitus constitute the **volsella** (vol Figs. 15B, 17B). The **penisvalva** (pen Figs. 15B, 17B), protruding slightly beyond the tip of the digitus, is attached to the stick-like **valvura** (vvu Fig. 17B), which acts as a helping lever. The gonoforceps and the penisvalva, together with the volsella, form the main parts of the external **phallus** (pha Fig. 15B).

Ventrally, the parossiculi abut medially, forming a distinct median fold (Fig. 15B). Submedially, adjacent to the fold, the parossiculi are distinctly strengthened. Close to the fold, each parossiculus is furnished with three short, stout, peg-like sensillae. Apically, the parossiculi are drawn out into a median, semi-triangular process.

##### Biosteres carbonarius

The cupula (cup Figs. 16B, 17D) in *B. carbonarius* is more or less obtusely crescent-shaped and it is much wider than it is long. The parossiculus (par Figs. 16B, 17D) is membranous and poorly defined. The more sclerotized and warped, cone-shaped digitus (dig Figs. 16B, 17D), with its concave lateral margin and convex mesal margin, is glabrous and less than one third the size of the gonoforceps (gon Figs. 16B, 17D). The penisvalva (pen Figs. 16B, 17D) is longer and more pointed than in *O. dissitus*.

## Discussion

### Terminology

Based on hypothesized homologies between braconid structures and structures in other hymen opterans, we have derived a set of recommended terms for braconids. These terms and their definitions were added to the Hymenoptera Anatomical Ontology (HAO) (http://hymao.org), if not present, and the terms are hyperlinked to the HAO from the tables in the Appendix S1. New terms or definitions proposed here are followed by an asterisk.

#### Acetabulum

Ronquist and Nordlander [25] proposed the term “fossa” or “articular fossa” for the socket of a ball-and-socket joint. More generally in entomology, the term “acetabulum” is used for the same concept [64], which earlier also have been in use by hymenopterists such as e.g. both Duncan [23] and Michener [24]. To align hymenopteran terminology better with that used in other insect groups, we propose to change the label for a fossa sensu Ronquist and Nordlander [25] to “acetabulum”. This has consequences for the naming of the anteroventral region of the mesopectus in Apocrita, currently referred to as the “acetabulum”. See “hypocnemium”.

*Face* (fce Figs. 1A–B, 1D, 2A). The region of the cranium that extends from the median ocellus (ocl Figs. 1A–B, 1D, 2A–B) to the clypeus (cly Figs. 1A–B, 2A). Terms for the anterior regions of the cranium vary considerably among authors. Snodgrass [69], Pratt [13], Chapman [70], Gullan and Cranston [71], and Grimaldi and Engel [72] use the term “frons”, which they define as the median, unpaired region (“unpaired sclerite”) between the compound eyes, extending from the epistomal sulcus (between the two anterior tentorial pits) up to the median ocellus. Many hymenopterists use “frons” for a smaller region, namely the part of the cranium between the antennal sockets and the median ocellus, while the region between the epistomal sulcus (esu Figs. 1A–B) and the antennal sockets is referred to as the “face” [2], [9], [11], [13], [73]. Other hymenopterists refer to the regions above and below the antennal sockets as the “upper face” and “lower face”, respectively, while the term “face” is used collectively for the lower and upper face, sometimes including the clypeus [19], [20], [25].

We suggest using the term “frons” for the entire region from the epistomal sulcus and up to the median ocellus, delimited laterally by the compound eye and the malar groove. The term “face” is best interpreted as a synonym of frons, but the latter term is preferable because it agrees with the terminology used for other insects. “Lower face” and “upper face” can then be used to refer to the part of the frons (or face) below and above the antennal sockets, respectively. Note that the clypeus is not part of the lower face or the frons (face) according to these definitions.

*Occipital condyle* (oco Fig. 2C). The lateral postoccipital lobe on the postero-dorsal margin of the occipital foramen, with which the anterior end of the cervical prominence articulates, was called the “odontoid” (plural “odontoidea”) by Ross [74]. Linguistically, “odontoid” is an unusual construct because it is possible to treat this Greek derivation as both an adjective (tooth-like) and a noun (something tooth-like). However, similar usage is well established in vertebrate anatomy. For instance, “hyoid” is used as a noun referring to the hyoid bone. The term “occipital condyle” is also commonly used, e.g. [22], [23], [64], [75]; often, it has been unclear whether the term is meant to refer to the entire lobe or just the articular point, which is positioned on the posterodorsal corner of the lobe. To clearly distinguish between these structures, Ronquist and Nordlander [25] used “odontoid” (although they incorrectly used the plural form as if it were the singular) for the lobe and “occipital condyle” for just the articular point. In practice, however, there is rarely a need to distinguish between the two, so we consider it acceptable to use “occipital condyle” for the entire postoccipital lobe, including the articular point, and this is the usage we follow here.

*Malar groove* (mgr Figs. 1A, 2A). A groove from the eye to the ventral margin of the cranium is often present in the Hymenoptera. It is variously referred to as the “malar suture” [2], [8], “malar sulcus” [20], [21], [35], [64] or “malar groove” (e.g., [76]). Because it is unclear whether this structure is a suture, sulcus or impression according to the usual definitions of these terms [19], [25], we prefer the more descriptive term “malar groove”, which can be applied across taxa without study of internal anatomy or ontogenetic development.

*Gula* (gul Fig. 2C). The term “gula” was introduced for an area on the posterior side of the cranium in apocritan Hymenoptera by Ronquist and Nordlander [25]. The area lies between the occipital and oral foramina, and is bordered laterally in *Ibalia* by the sulcus (“gular sulcus” of Ronquist and Nordlander [25]) corresponding to the ventral, ridge-like extension of the posterior tentorial arm (pts Fig. 1C). In *Ibalia*, the interior ridge-like extensions meet medially about half-way to the oral foramen and continue as a single ridge before ending ventrally near the submedially placed condyles of the cardines [25]. Externally, the gula narrows to a median strip about half way to the hypostoma (hst Figs. 2B–C). The microsculpture of the gula is usually distinctly different from the surrounding postgenal and hypostomal regions of the cranium, appearing densely pubescent or heavily wrinkled, especially along the midline. The peculiarly sculptured and pubescent median area appears to be present across a broad range of hymenopteran taxa, including taxa where the bordering ridges are absent as far as can be judged from external structure.

The area is analogous to the gula in other insects, such as prognathous Coleoptera, in that it lies between the oral and occipital foramina, and in that it is bordered laterally by lines and ridges that connect to the posterior tentorial pits (e.g. [69], Fig. 68; note, however, that the lines/ridges are dorsal to the posterior tentorial pits in prognathous Coleoptera, and ventral to the pits in Ichneumonoidea and other Hymenoptera). For these reasons, Ronquist and Nordlander [25] considered the use of the terms “gula”, “gular sulci”, and “gular ridges” justified for the area and its bordering sulci/ridges. The terms “gula” and “gular ridges” have since been widely adopted in the cynipoid literature, e.g. [77], [78], [79] and they are also used in the Chalcidoidea [80].

There is a number of other terms that have been applied to the sclerotized region between the occipital and oral foramina in Hymenoptera, including “postgenal bridge”, “hypostomal bridge” and “postoccipital bridge” [35], [74], [81]. However, these terms are typically used for cranial regions that lie largely outside the gula sensu Ronquist and Nordlander [25], or they are used to describe conditions that are independent of the presence or absence of the gula. For instance, the “hypostomal bridge” is defined as the median area between the hypostomal carinae (hca Figs. 2B–C), which is a much larger region than the gula sensu Ronquist and Nordlander [25] in many Apocrita, including the opiines described here as well as some Chalcidoidea [80]. The hypostomal bridge, when defined, typically includes a more or less distinct median gula.

In *Ibalia* and a range of other Apocrita, in contrast, the hypostomal carinae do not continue dorsally, making it difficult to distinguish a hypostomal bridge at all [25]. Particularly when the gula is largely reduced to a median strip, such taxa are said to have a “postgenal bridge”. However, the latter term is typically understood to refer to the medially expanded postgenal regions rather than to the gula itself; see, e.g., illustration in Gibson et al. [80]. Furthermore, when a hypostomal bridge is present, it is typically regarded as replacing the postgenal bridge altogether, whereas the gula sensu Ronquist and Nordlander [25] is present regardless of whether the closure between the oral and occipital foramina can be classified as hypostomal or postgenal. A “postoccipital bridge” has been described in several sawflies, including the Cephidae, but it is an internal structure formed by the median fusion of the attachments of the ventral profurco-postoccipital muscles [81], and hence not homologous to the gula sensu Ronquist and Nordlander [25].

Given that the term gula now appears to be well established in the Cynipoidea and Chalcidoidea, we see no need to change it because of the mismatch with respect to one of Snodgrass’s original topological criteria; the position relative to the tentorial pits.

Mandibular lancea* (mla Figs. 4B–D). A spear-like ventral flange on the mandible (Figs. 4B–F, mnd Figs. 1A–B, 2A), issuing from the mandibular condyle (mco Figs. 4B–D) and ending apically in a more or less sharp point. This structure appears in many opiines and has previously been referred to as the “basal mandibular tooth” [2], [52], [82], [83]. However, the lancea is very distinctly separated from the ordinary teeth and its structure is conspicuously different.

Sternacostal carina* (scc Figs. 7A, 10B–C). A transverse carina on the prosternum (pst Fig. 7A, 10B), between the probasisternum (pbs Fig. 7A, 10B ) and profurcasternum (pfs Figs. 7A, 10B–C). Many hymenopterans appear to have an invagination in a similar position, e.g. the prosternal groove [5], sternacostal suture [69] or the prosternal incision [35].

Anteromesoscutum* (ams Figs. 5A, 6A, 9A). The mesonotal sclerite anterior to the transscutal articulation ( = mesoscutal sclerite *sensu* Ronquist and Nordlander [25].

*Medio-posterior mesoscutal depression* (mmd Fig. 6B). In braconids, it is often a more or less pit- or groove-like depression where the notauli (not Figs. 5A, 6A, 9A) join posteriorly. Fischer [52] named it “Rücken-grübchen”, Achterberg [84]“mesoscutal pit”, and Wharton et. al [2] “mid pit”. In more recent publications, Achterberg [10] refers to it as the “medio-posterior mesoscutal depression”, which is the term we recommend here. The structure is apparently homologous to the median mesoscutal impression sensu Ronquist and Nordlander [25] in cynipoids but probably not to the median mesoscutal sulcus [30], which typically starts at the anterior margin of the mesoscutum (msc Figs. 5A, 6A) and rarely reaches the posterior margin.

*Subalar tubercle* (sat Fig. 5A). A small articular process posterodorsally on the subalar area. It is apparently this process that is equipped apically with a cup-shaped socket that receives the second axillary sclerite during the upstroke of the wing in the honey bee [85].

Triangular axillar region* (tax Fig. 6A) The region that is the lateral surface of the dorsal axillar area (daa Figs. 5A, 6A), defined laterally by the axillar carina (axc Figs. 5A, 6A, 9C) and mesally by a proposed line formed by the anterior prolongation of the lateral margin of the mesoscutellar disc.

*Mesoscutellar disc* (msd Figs. 5A, 6A, 9A, 10A). The dorsal area of the mesoscutellum (mum Fig. 5A, 6A) that is delimited anteriorly by the scutoscutellar sulcus and laterally by the mesoscutellar trough (sct Figs. 5A, 6A) and that internally houses the pulsatory organ of the fore wing.

Mesoscutellar trough* (sct Figs. 5A, 6A). The depressed area on the side of the mesoscutellum, ending anteriorly in the mesoscutellar pit, was termed the “axillula” by Ronquist and Nordlander [25], a term that is fairly widely used in the Apocrita e.g. [35], [80]. However, in the Ichneumonoidea in particular, it is fairly clear that this area is serially homologous to a metanotal area that has been referred to as the “axillary trough of the metanotum” [13] or “metanotal trough” [25], [35]. Townes [14] also used the term “axillary trough of mesonotum” for the axillula of other authors. We suggest here that the terminology be based on the serial homology, and the two areas referred to as the mesoscutellar and metascutellar troughs, respectively.

Mesoscutellar pit* (mpi Fig. 6A). The deep, apophysis-marking pit situated anterolaterally of the mesoscutellar trough (sct Figs. 5A, 6A), just mesal to the sclerite’s keel-like postalar process (pap Figs. 5A, 6A).

*Posterior bar of mesoscutellum* (pbm Fig. 6A). To reflect the change from axillula to mesoscutellar trough for the posterolateral mesoscutal impression, we propose to change the name of the bar beneath the impression from subaxillular bar [25] to posterior bar of mesoscutellum.

*Hypocnemium* (hum Figs. 5A, 7A). “Prepectus”, “acetabulum” and “epicnemium” are all terms used in the Apocrita for the anterolateral and anteroventral portions of the mesopectus. The prepectus, it is now well established, is primitively an intersegmental sclerite, positioned between the prothorax and mesothorax [30]. It differs widely in length and size in the extant Hymenoptera but is characterized by bearing the occlusor muscle of the anterior thoracic spiracle. A free prepectus has been retained in most Symphyta, but beside Chalcidoidea in few parasitic wasps. According to available evidence, particularly the position of the spiracular occlusor muscle, the prepectus has either been lost or been incorporated in the pronotum in the Braconidae. The prepectus is rarely incorporated in the mesopectus in Apocrita [30], [66]. Hence, “prepectus” is not an appropriate term for the anteroventral portion of the mesopectus in Braconidae, nor in most other Apocrita.

In numerous species of Braconidae, there is a carina on the posterior part of the mesopectus termed “the postpectal carina” due to its location just anterior to the middle legs. The use of the term “prepectal carina” for the carina just posterior to the fore legs, on the opposite portion of the mesopectus, is popular in this group e.g. [8], [9], seemingly because “prepectal” is an antonym to “postpectal”. However, for the reasons stated above, it is an unfortunate use of terms and should be discouraged.

In many apocritans, “acetabular carina” is used for the carina bordering the mesopectal area that accommodates the posterior surfaces of the procoxae e.g. [25], [35], [86]. The area itself should hence be referred to as the “acetabulum”. In the Ichneumonoidea in particular, the terms “epicnemium” and “epicnemial carina” are used for a similar but larger portion of the anterior and ventral parts of the mesopectus [2], [13]. In addition to the acetabulum, the epicnemium typically includes also the anterior portion of the lateral surface of the mesopectus. While the acetabular carina is strongly curved and ends anteriorly close to the posteroventral corner of the pronotum, the epicnemial carina is straight and extends farther dorsally.

Some Sphecidae sensu lato have both a complete acetabular carina and a so-called omaulus, which appears to be structurally and positionally analogous, if not homologous, to the epicnemial carina [86]. Other sphecids and ichneumonoids lack a complete acetabular carina but have a medioventral posterior bend or ω-shaped double bend in the epicnemial carina, apparently corresponding to the remnants of the median section of the acetabular carina [19], [86]; see also Morphbank collection http://morphbank.net/?id=579716. In the Chalcidoidea, the anteroventral area of the mesopectus is described by Gibson et al. [80] as being defined laterally by the epicnemial carina and posteromedially by the acetabular carina, again suggesting that the area is a composite of the epicnemium and acetabulum.

Unfortunately, “acetabulum” is defined more generally in insects as the cavity housing the condyle of an articulation [64]. To avoid confusion in comparisons across insect orders, we suggest that “acetabulum” be used in the more generally accepted sense also in the Hymenoptera (see above), while we propose the new term “hypocnemium” for the area defined by the acetabular carina sensu Bohart and Menke [86]. The term “hypocnemium” refers to the fact that the area constitutes the lower (*hypo*- is Greek for under; *cnemis* is Latin and refers to the shin) part of the epicnemium. The acetabular carina, when present and clearly distinguishable from the epicnemial carina, should hence be termed the “hypocnemial carina”.

*Mesepimeral sulcus* (msu Figs. 5A). In ichneumonoids, there is almost always a distinct sulcus running close to the posterior margin of the lateral surface of the mesopectus, i.e. from the mesocoxa towards the pleural wing process. The sulcus is the external sign of a very strong internal ridge (mer Figures 7E, 8A, 8D, 10A, 10B). The external line has commonly been referred to as the mesopleural sulcus [8], [9], [19] or suture [13] because it is analogous to the mesopleural sulcus in sawflies and other insects. However, muscle attachments suggest that the sulcus in ichneumonoids and other apocritans is largely a secondary structure formed posterior to the primitive mesopleural sulcus, on the mesepimeron [25], [66], [87]. Therefore, this structure in the Ichneumonoidea should be called the mesepimeral sulcus and not the mesopleural sulcus.

Mesepimeral flange* (mef Fig. 5A). The posterior area of the epimeron, behind the mesepimeral sulcus.

Mesepimeral lobe* (mlo Fig. 5A). The posterodorsal mesepimeral lobe covering the posterior thoracic spiracle.

Subalar bridge* (sub Fig. 5A). A reinforcement beneath the subalar impression (sai Fig. 5A), which connects the mesepimeral flange to the mesepisternum (mes Figs. 5A, 6A, 7A).

*Mesopleural scrobe* (mpb Figs. 5A, 10A). Many ichneumonoids have a distinct, slit-like, short horizontal sulcus posteriorly on the lateral surface of the mesopectus. A pit-like structure, which may lie in the middle but more typically close to the anterior end of this sulcus, is usually referred to as the “episternal scrobe” [2], [8], [19], [24], [64], [86]. Townes [14] called it “mesopleural pit” and also recently, it has been referred to as the “mesopleural pit” [35], [66]. Internally, the sulcus and pit correspond to the mesopleural apodeme (map Figs. 9A, 9D), serving as the site of origin of the mesopleuro-mesocoxal and the second mesopleural-metanotal muscles [66]. The slit-like sulcus is likely to be a short remnant of the primitive mesopleural sulcus defining the boundary between the mesepisternum and the mesepimeron (see mesepimeral sulcus; see also [66]). Therefore, it is unfortunate to use a name identifying this structure as episternal and we instead suggest calling it the “mesopleural scrobe”. In some taxa may neither the sulcus nor the pit be visible at all, although the mesopleural apodeme is presumably always present.

Since the mesopleural scrobe seems to be a part of the mesopleural sulcus, the latter term could also be used. However, the mesopleural scrobe is a very distinct part of the mesopleural sulcus, which is present in many Apocrita, and a separate term for this structure therefore seems warranted, especially since the interpretation of the mesopleural sulcus in Apocrita is not straightforward.

*Metascutellar disc* (med Figs. 5A, 6A, 10A). “Postscutellum” [13], [52], “metascutellum” [25], [34], [35], [66] and “dorsellum” [73], [80], [88], [89] are all in use for the same region in the Apocrita; a small raised structure medially on the metanotum. However, “postscutellum” is a feature of the mesopostnotum in Symphyta, which is not homologous to the part called “postscutellum” in Apocrita. Therefore, this term should be avoided in the Apocrita. “Dorsellum” is widely used for the elevated metanotal structure in the Chalcidoidea [80], [88] as well as in the Cynipoidea. e.g. [89]. It is a descriptive term; “dorsellum” is the diminutive form of “dorsum” in Latin and means “small back”. Ronquist [89] chose to use “dorsellum” over “metascutellum” because he considered it uncertain whether the elevated region was truly homologous to the mesoscutellum. Recent morphological research [35], [66] indicates that the region is likely to be homologous not to the entire mesoscutellum but to the mesoscutellar disc. Both the mesoscutellar disc and the median metanotal structure are elevated, and both internally house the dorsomedian pulsatile organ, pumping haemocoel out of the wings [66]. The entire mesoscutellum appears to be serially homologous to a larger part of the metanotum, including the metascutellar troughs. For these reasons, we propose the term “metascutellar disc” for the elevated median part of the metanotum.

Metascutellar trough* (met Fig. 6A). The depressed area lateral to the metascutellar disc (med Figs. 5A, 6A, 10A) has previously been referred to as the “axillar trough” [13], “lateral panel of metanotum” [80] or, most commonly, “metanotal trough” [25], [35]. Because it is likely to be a metascutellar structure, we propose to rename it *metascutellar trough*, clearly indicating the serial homology with the mesoscutellar trough.

Metanotal pit* (mnp Fig. 6A). The deep, apophysis-marking pit situated anterolaterally of the metascutellar trough.

Posterior bar of metascutellum* (pba Fig. 6A). The raised bar, running along the posterior margin of the metanotum, posterior the metascutellar trough. Apparently serially homologous with the posterior bar of the mesoscutellum.

Median metanotal notch* (mnn Fig. 6A). A small medial indentation at the posterior margin of the metanotum (mtn Figs. 5A, 6A, 9A, 10A).

Metapleural scrobe* (mtb Fig. 5A). A horizontal sulcus anterolaterally on the metapectal-propodeal complex. The sulcus may possibly be part of the primitive metapleural sulcus (mts Figs. 5A, 6A, 10A) extending from the metacoxal condyle to the metapleural wing process in most other insects, for instance in nearly all Symphyta. It corresponds internally to the metapleural apodeme [66], which is either fused with the lateral metafurcal arm or connected to it through muscles, as in the species examined here (Fig. 10E). We suggest a term that indicates serial homology with the mesopleural scrobe. Both structures correspond to internal apodemes that connect to the furca, and may represent modified portions of the primitive pleural ridge.

*Metasubpleuron* (spl Figs. 7A, 8A-B, 9A, 10A, 10E). The ventral region of the metapectus [24], [25].

Metapectal-propodeal rim* (mpr Figs. 7A, 10A). The reinforced rim that surrounds the propodeal and metacoxal foramina.

Basicoxal foramen* (bcf Figs. 11C). The proximal foramen of the coxa, through which muscles and other internal organs pass between the leg and the thorax. Because the term “coxal foramen” is commonly used for the corresponding foramen in the thorax, a separate term is needed for this structure.

Basicoxal girdle* (bag Figs. 11A-D). The raised annular girdle surrounding the basicoxal foramen.

Procoxal process* (pcp Fig. 11A). The lateral process of the procoxa articulating distally with the coxal condyle of the propleuron.

Mesocoxal process* (mcp Fig. 11B, 11D). The lateral process of the mesocoxa articulating distally with the coxal condyle of the mesopectus.

Metacoxal process* (mxp Figs. 11C). The lateral process of the metacoxa articulating distally with the coxal condyle of the metapectal-propodeal complex.

Procoxal pit* (cop Fig. 11A). The basal pit on the procoxal process (pcp Fig. 11A).

Basicoxal acetabulum* (bca Figs. 11B-D). The acetabulum on the basal part of the meso- and metacoxa, articulating with the coxal condyle of the pleuron.

##### Laterotergite

The lateral parts of each of the abdominal terga 2 to 7 (T2 – T7 Figs. 14A, 15A, 16A–B) are separated from the mediotergite by a more or less distinct gastral fold (gaf Figs. 14A, 15A) in ichneumonoids. The original term for the separated lateral part appears to be “laterotergite” e.g. [11], [21], [69], [90]. In Ichneumonoidea, this structure has often been referred to as the “epipleurum” [14], [91] or “epipleuron” [92]. This is unfortunate because the tergite is not of pleural origin, nor is it clear that it covers primitively pleural regions of the abdomen. Furthermore, the term “epipleuron” is used for a different structure in the Coleoptera [64]. More recently, braconid workers have started using the anglified version “lateral tergite” [2]. However, there is a disadvantage in using this phrase as a name of a specific structure, as it becomes unclear, without further context, whether one is referring to a tergite positioned laterally or the specific structure called “lateral tergite”. For this reason, and because we tend to prefer older, well-established terms in the name of terminological stability, we recommend the form “laterotergite”.

*Mediotergite* (T(X) Figs. 14A, 15A, 16A–B). The median sclerite of abdominal terga 2 to 7 is sometimes called the “median tergite” by braconid workers e.g. [2]. We prefer “mediotergite” because it is consistent with “laterotergite”, it is shorter, and it avoids the ambiguity between the reference to a specific structure and a simple descriptive expression (see “laterotergite”).

Gastral fold* (gaf Figs. 14A, 15A). A more or less distinct lateral longitudinal crease, ridge or fold on abdominal terga 2 to 7, defining the border between the mediotergite and laterotergite.

Petiolar collar* (pec Figs. 13A, 13F). The obliquely dorsally and laterally projecting anterior flange of the petiolar tergum, bearing the petiolar condyles (pco Figs. 13A–C, 13E–G, 14A, 15A) laterally.

Anterior petiolar process* (app Figs. 13A, 13D, 13F). The dorsal process on the anterior margin of the petiole (pet Figs. 13A–F, 14A, 15A), which fits into the small dorsal section of the propodeal foramen (pfo Fig. 10A) above the propodeal teeth (ppt Fig. 10A).

Petiolar fovea* (pef Fig. 13A). A lateral depression on the dorsal surface of the petiolar collar (pec Figs. 13A, 13F).

*Dorsal petiolar carina* (dpc Figs. 13A–B, 13D–F). A longitudinal carina on the petiole, running from the petiolar collar upwards, and then continuing longitudinally along the dorsolateral margin of the petiole. This carina was called the “dorsal longitudinal carina” by Sharkey in [2] but “dorsal carina” is more commonly used in braconids [2], [8], [9]. In ichneumonids, Townes [13] used the term “dorsolateral carina” for an apparently homologous structure. The dorsope (dor Figs. 13D–F), when present, is situated at the proximal end, between the dorsal and lateral petiolar carinae.

Lateral petiolar carina*. (lpc Figs. 13B, 13E–F). A longitudinal carina running from the petiolar spiracle and towards the petiolar collar along the lateral margin of the petiole. It marks the dorsal margin of the glymma (gly Figs. 13B) and laterope (lat Figs. 13B), when present.

Lateropal apophysis* (lap Fig. 13C). The apophysis marked externally by the laterope (lat Figs. 13B).

Posterior area of second sternum* (S2b Figs. 14A, 15A). The membranous posterior area of the second abdominal (petiolar) sternum.

*Epipygium* (epi Figs. 14A, 15A, 16A). The syntergum composed of the primitive abdominal tergites and appendages (the cerci) posterior to abdominal tergite 8.

### Conclusions

The taxonomic status of one of the studied species, *Biosteres carbonarius* (Nees, 1834), is somewhat uncertain. Achterberg [93] considered it as conspecific with *B. impressus* (Wesmael, 1835), as the females of these two species are identical morphologically, as far as known. However, the males differ conspicuously in the coloration of the metasoma. In *B. carbonarius*, the male metasoma is entirely black while in *B. impressus* it has a contrasting transverse band of varying width and color (from yellowish to dark brownish) on each of the median abdominal tergites 4 – 7. Both male color forms have been reared from the same material [93] but this does not in itself prove that the forms are conspecific. We do not take a position here on whether *B. carbonarius* and *B. impressus* are distinct species but we would like to point out that all the specimens studied by us and described here belong to the entirely black male form, which is the only form recorded thus far from the catches of the Swedish Malaise Trap Project [94].

There is surprisingly little sexual dimorphism in the examined species except for the metasoma. For instance, Fisher [52] indicated that opiine males in general have more flagellomeres (fla Figs. 2A, 3A–B) than females but we did not find any significant differences between the sexes of the examined species. For instance, in *O. dissitus* we found that females had 18.9±1.3 flagellomeres (n = 30) while males had 18.8±1.7 flagellomeres (n = 48). Thus, the number of flagellomeres varies more in males than in females, but the average number is the same in both sexes.

The number and distribution of antennal multiporous plates is commonly thought to vary between sexes e.g. [19] but we did not find this to be the case in the studied species. There is significant variation in multiporous plates related to the size of the specimen but this variation overshadows any differences between sexes.

In both *O. dissitus* and *B. carbonarius*, males have slightly larger eyes than females, which is a common condition among braconid wasps [42]. It is important to take this into account when studying characters related to the eyes, e.g., the length of the malar groove (mgr Figs. 1A, 2A).

Both Opiinae and Alysiinae are usually considered to be cyclostome braconids. Cyclostomes are generally characterized by the ventral margin of the clypeus being distinctly concave, leaving an oval opening above the dorsal margin of the closed mandibles. Furthermore, the ventral margin of the clypeus is typically inflected and the labrum (lab Fig. 1A) distinctly concave, both the latter surfaces being polished. Although opiines have a similar appearance, they lack the polished inflected part of the clypeus, most species have a flat and setose labrum, and there is not always a distinct oval opening above the mandibles (see i.e. Fig. 2E).

The two species studied here illustrate some of the variation among opiines quite well. In *B. carbonarius*, the stout mandibles regularly close the space beneath the clypeus, which has a straight or slightly convex ventral margin. In *O. dissitus*, on the other hand, there is occasionally an elliptic gap between the ventral margin of the clypeus and the mandibles, leaving the labrum more or less exposed. In general, there appears to be considerable variation among opiines in this region, some of which might be phylogenetically informative.

Several members of the Opiinae have a distinct mandibular lancea (mla Figs. 4B–D) on the ventral margin of the mandible (Figs. 4B–F, mnd Figs. 1A–B, 2A), previously referred to as the “basal mandibular tooth” [2], [82], [83] or the “ventral carina of the mandible” [52]. The lancea is present in both of the species studied here. It is more prominent in *O. dissitus* than in *B. carbonarius,* but because the mandible is more tilted ventrally in the former, it is actually more difficult to discern the lancea in this species without dissection. The lancea is considered to be absent in some opiines. It is still unclear whether presence of the lancea is a synapomorphy of all opiines, in which case it must have been secondarily lost in some groups, or a synapomorphy of a subgroup of opiines, in which case the lancea would be primitively absent in some groups.

The function of the lancea is unknown but just beneath the apex of the lancea, the margin of the mandible appears sharp, suggesting that the lancea is used to capture and cut through thin sheets of tough fabric. Thus, it appears possible that the lancea is used to exit the puparium of the host larva, which is much tougher than the cocoons constructed by braconids. It is commonly thought that alysiines, which are also dipteran parasitoids, have evolved their exodont mandibles to solve the same problem [2].

Some braconid subfamilies have a complete occipital carina (oca Figs. 2A–B) while others lack it completely. Opiines are unusual, however, in that the condition varies among members. The most common state is to have a partial occipital carina, like both species studied here, but it is unclear whether this is primitive for the subfamily. Whether or not the occipital carina ventrally joins the hypostomal carina is another character in this region that is often phylogenetically informative. In most opiines, the carinae are separated ventrally as in *O. dissitus* and *B. carbonarius*, but there are a few exceptions, e.g., among species of *Opius*.

Many opiines have a distinct pronope (pnp Figs. 6C, 9B), unlike *O. dissitus*. However, it is unusual that it is as large as the pronope in *B. carbonarius*; in fact, the large pronope is one of several characteristic features of the genus *Biosteres*.

Opiines in general lack the epicnemial and hypocnemial carinae, as do both of the species examined here. However, the hypocnemium (hum Figs. 5A, 7A) is still visibly separated from the surrounding mesopectal regions by differences in sculpture, pubescence, and surface angles, indicating that the hypocnemial carina was lost secondarily.

There is significant variation in the shape of the fore wing vein 3RS in the Opiinae. Its first abscissa, 3RSa, is almost equal in length to 2RS in Biosterini *sensu lato*, whilst it is noticeably longer than 2RS in Opiini *sensu lato.* Thus, the differences between the studied species in this character seem to be of phylogenetic importance. The second abscissa of 3RS, 3RSb, is long and curved in the two species studied here but it varies considerably in the subfamily. For instance, it can be long and straight as in *Diachasmimorpha* spp., or short and not reaching the wing margin as in *Ademon spp*. The phylogenetic significance of this variation is still unclear.

Basibuyuk and Quicke [36] reported the presence of paddle-shaped setae on the basitarsus, anterior to the strigilar comb, in a number of hymenopteran lineages. However, they failed to report similar setae posterior to the comb despite the fact that they examined a number of braconid representatives in addition to numerous other hymenopterans. Thus, it is possible that the two paddle-shaped setae we found posterior to the strigilar comb in both the examined opiines represent a novel feature derived within this group. In any case, the occurrence of these setae among opiines and related braconids clearly warrants further scrutiny. We did not find any differences between the sexes in the structure of the paddle-shaped setae, suggesting that they do not play any role in courtship. Since they are located on the posterior side of the strigil, and thus make contact with the antenna before the dirt on the latter is scraped off by the strigil, it is possible that they secrete some kind of “cleaning fluid” that facilitates or improves the cleaning. We were not able to detect any traces of secretions on the setae, but the specimens we used for SEM studies were cleaned in strong solvents that could have removed such traces. Regardless of whether they have a secretory function, they probably contribute mechanically to the cleaning of the antenna.

When hymenopterans clean their wings, the hind leg distad of the femoro-tibial joint is pushed backward over the wing surface e.g. [5], [23], [95]. To improve the cleaning of the wing, this part of the hind leg is often more densely pubescent among Apocrita, as was the case in both species examined here.

In braconids, it is common that the petiole is strengthened anteriorly by convoluted cuticle forming deep pits and various ridge-like structures externally. In opiines, the lateral, longitudinal petiolar impression called the glymma (gly Figs. 13B) typically ends anteriorly in a deep laterope [96], as in *O. dissitus,* while the dorsope is lacking. In *B. carbonarius*, however, the glymma and laterope are missing and replaced by a pair of huge dorsopes on the dorsal surface, a condition characteristic for the genus *Biosteres*. Apparently, opiines either have lateropes or dorsopes, never both (unpublished data). It is unclear why this should be the case since other braconids, e.g. Meteorinae, have both structures. Regardless of the reasons for this pattern, it is clear that these petiolar structures are potentially phylogenetically informative in opiines.

Many apocritans have a pair of distinct propodeal teeth situated dorsolaterally on the margin of the propodeal foramen. It has generally been assumed that the mesosomal acetabula articulating with the petiole are situated on these teeth (e.g. [66]). In the examined opiines, however, we found that the petiole actually articulates with processes (pac Fig. 10A) that are situated beneath the propodeal teeth (ppt Fig. 10A), on the lateral margin of the propodeal foramen (pfo Fig. 10A). These acetabula articulate with the strengthened dorsolateral corners of the petiole. Medially, the petiole is equipped with a distinct process, the anterior petiolar process (app Figs. 13A, 13D, 13F), which fits inside the dorsal part of the propodeal foramen above the propodeal teeth. The teeth themselves fit underneath the head of the process. Apparently, the anterior petiolar process and the propodeal teeth serve to stabilize the metasoma laterally when it is moved by the mesosomal muscles attached to the petiole. It would be interesting to examine this articulation in more detail across a broader selection of taxa to find out whether the structure we found in opiines is more generally present in other taxa with propodeal teeth, or whether this condition represents an exception.

Consistency in the terminology used to describe various morphological features is crucial for efficient communication. This is particularly true when working with such an immensely diverse and poorly known group as the Ichneumonoidea in which just a fraction of all species has been scientifically described. Inconsistent terminology may result in false hypotheses of homology, leading to erroneous phylogenetic interpretations, and in incorrect taxonomic conclusions, causing unnecessary proliferation of nomenclatural synonyms. For instance, if the same term is used for analogous but independently derived structures, they may be mistakenly believed to be homologous and indicate close relationship. Similarly, homologous structures may be misidentified as homoplasies if they are named differently by different specialists.

We believe that the selection of terms for morphological structures should be based primarily on hypotheses of homology. It is also important that the original meaning of the chosen term, often a word of Greek or Latin origin, matches the structure, for which it is used. In some cases, we believe that it is also permissible to name functionally or structurally analogous structures using the same term. For instance, it seems justifiable to use the terms “wing” and “head” in insects, even though these structures are not homologous, only analogous, to similarly named structures in other animals.

The terminology section above details the justification for the more controversial aspects of the terminology we propose for ichneumonoids in general and opiine braconids in particular. To make our terminology widely available, we present it both in this paper and in the online Hymenoptera Anatomical Ontology database (http://hymao.org), making it possible to link to our terminology both from species descriptions and from lists of morphological characters used in phylogenetic analyses. Further discussion of our terminology is welcomed as contributions to the Hymenoptera Anatomical Ontology project.

Since the vast majority of all insect species have not yet been described, systematic entomologists in particular are faced with a difficult task in responding to recent calls to complete the inventory of the planet’s biota in a relatively short time period. Although molecular methods are increasingly being used in this quest, there is no doubt that external morphology will continue to play an prominent role in discovering, circumscribing and describing new insect species. Our hope is that this work will contribute to developing a robust platform for such diversity-related morphological studies of braconids, representing one of the richest species radiations on our planet.

## Supporting Information

Appendix S1 **List of anatomical terms, with definitions and figure references.** Terms are hyperlinked to entries in HAO, where applicable. Hyperlinks to HAO have the structure http://purl.obolibrary.org/obo/HAO_XXXXXXX, where XXXXXXX is the HAO ID number. New terms or definitions proposed here are written in italic and followed by an asterisk.
(DOC)
