# Supplementary material for: Skeletal Morphology of Opius dissitus and Biosteres carbonarius (Hymenoptera: Braconidae), with a Discussion of Terminology
Source: PLoS One. 2012 Apr 30;7(4):e32573. doi: 10.1371/journal.pone.0032573 (PMC3340384; doi:10.1371/journal.pone.0032573)
Supplement: Appendix S1 — List of anatomical terms, with definitions and figure references. Terms are hyperlinked to entries in HAO, where applicable. Hyperlinks to HAO have the structure http://purl.obolibrary.org/obo/HAO_XXXXXXX, where XXXXXXX is the HAO ID number. New terms or definitions proposed here are written in italic and followed by an asterisk. (DOC) [file pone.0032573.s001.doc]

| **Abbr.** | **Term [HAO ID]** | **Notes** | **Figures** |
| --- | --- | --- | --- |
| aap | antennal articular process | The basal, condyle-like proximal process of the radicle, which articulates with the antennifer of the antennal rim. | 3B |
| afo | antennal foramen [0001022] | The foramen that is located on the head, and into which the radicle is inserted. | 1A-B, 1D |
| amm | anteroventral mesopectal margin | The anteroventral mesopectal margin that is folded into a reversed, spout-like, bowed rim. | 7A, 9A-B, 10A, 10D |
| ams | Anteromesoscutum  [0001490] | The mesonotal area that is located anterior to the transscutal articulation. | 5A, 6A, 10A |
| ann | annellus  [0000095] | The ring-like area that is located proximally on the first flagellomere, and that is separated from the latter by a complete or incomplete sulcus. | 2A, 3A-B |
| app | *anterior petiolar process** | The dorsal process on the anterior margin of the petiole, which fits into the small dorsal section of the propodeal foramen above the propodeal teeth. | 13A, 13D, 13F |
| apr | anteromedian pronotal ridge | The ridge that extends parallel to the anterior (ventral) margin of the pronotum and delimits the site of origin of the pronoto-propleural arm muscles. | 9A-B |
| apt | anterior process of the tentorial bridge | The anteriorly directed process of the tentorial bridge, which serves as the tendon for the posterior contractor of the pharynx. | 1C |
| ari | antennal rim  [0000103] | The rim that surrounds the antennal foramen. | 1A-B, 2A |
| aro | arolium  [0000148] | The pretarsal lobe that is connected proximodorsally with the manubrium and proximoventrally with the planta. | 12G-H |
| asc | antennal scrobe  [0001432] | The scrobe that is located dorsally of the antennal foramen and is for the reception of the antenna. | 1E-F |
| ata | anterior tentorial arm  [0001454] | The apodeme that extends between the anterior tentorial pit and the tentorial bridge. | 1C |
| atp | anterior tentorial pit  [0000126] | The tentorial pit that corresponds to the anterior end of the tentorium and partially delimits the clypeus. | 1A-C |
| axc | axillar carina  [0000156] | The line that separates the dorsal and lateral axillar surfaces. | 5A, 6A, 10C |
| axi | axilla  [0000155] | The area delimited anteriorly by the transscutal articulation and posteriorly by a virtual line along the scutoscutellar sulcus, including the mesoscutellar pit if present, to the mesonotal margin, but not including the postalar process if present. | 5A, 6A |
| bag | *basicoxal girdle** | The raised annular girdle surrounding the basicoxal foramen. | 12A-D |
| bca | *Basicoxal acetabulum**  [0001915] | The acetabulum on the basal part of the meso- and metacoxa, articulating with the coxal condyle of the pleuron. | 12B-D |
| bcf | *basicoxal foramen** | The proximal foramen of the coxa, through which muscles and other internal organs pass between the leg and the thorax. | 12C |
| bta | basitarsus | The tarsomere that is the most basal article of the tarsus, connected proximally with the tibia and distally with the second tarsomere via membranous conjunctivae. | 12A-C, 12E-F |
| btc | basitarsal comb  [0001180] | The row of setae that extends along the ventral margin of the probasitarsus. | 12E |
| cal | calcar  [0000185] | The modified protibial spur that is used to assist in cleaning the antennae. | 12E |
| cap | cervical apodeme  [0000192] | The apodeme that is located posteriorly on the cervical prominence and that serves as the site of insertion of laterocervical muscles. | 8C |
| car | cardo  [0000187] | The area that is located proximally on the maxilla, proximal to the stipes. | 4A |
| cer | cercus  [0000191] | The appendage that is located apicolaterally on one of the apicalmost abdominal terga. | 14A, 15A-B, 16A,17A, 17C |
| cey | compound eye  [0000217] | The compound organ that is composed of ommatidia. | 1A-B, 1D, 2A-B |
| cop | *procoxal pit** | The basal pit on the procoxal process. | 12A |
| clw | tarsal claw  [0000989] | The curved, claw-like sclerite that projects from the apex of the last tarsal segment on either side of the arolium of the pretarsus. | 12G-H |
| cly | clypeus  [0000212] | The anteromedial area of the cranium, which is the site of origin of the clypeo-epipharyngeal muscle of the head capsule, lying below the (lower) face, and to which the labrum is articulated. Dorsally usually separated from the (lower) face by an epistomal sulcus and laterally by the clypeo-pleurostomal lines. | 1A-B, 2A |
| cox | coxa  [0000228] | The proximal leg segment that is connected proximally to the thorax and distally to the trochanter via conjunctivae and muscles. | 5A, 8E, 12A-D |
| cpl | clypeo-pleurostomal line  [0000211] | The line that extends from the anterior tentorial pit to the cranial margin and separates the clypeus laterally from the pleurostoma. | 1A |
| cpr | cervical prominence  [0000193] | The articular process that is located on the anterior margin of the propleuron and that articulates anteriorly with the occipital condyle. | 7A, 8B-C |
| cup | cupula  [0000238] | The sclerite that is usually ring-like and is connected distally to the gonostipes and proximally to the abdominal sternum 9 via muscles and conjunctivae. | 16B, 17B,17D |
| daa | dorsal axillar area  [0000252] | The area that is located medially on the axilla and is delimited anteriorly by the transscutal articulation, laterally by the axillar carina and posteromedially by the scutoscutellar sulcus. | 5A, 6A |
| dig | digitus  [0000385] | The sclerite that is located distally on the parossiculus. | 15B, 16B, 17B, 17D |
| dor | dorsope  [0000280] | The dorsal pit located at the proximal end of the petiole, between the dorsal and lateral petiolar carinae. | 13D-F |
| dpc | dorsal petiolar carina | The longitudinal carina on the petiole, running from the petiolar collar upwards, and then continuing longitudinally along the dorsolateral margin of the petiole. | 13A-B, 13D-F |
| dta | dorsal tentorial arm  [0000275] | The apodeme that arises anterodorsally from the anterior tentorial arm and extends to the anterior wall of the cranium. | 1C |
| epi | epipygium | The syntergum composed of the primitive abdominal tergites posterior to abdominal tergite 8. | 14A, 15A, 16A |
| esu | epistomal sulcus  [0000306] | The sulcus that extends between the two anterior tentorial pits, partly separating the (lower) face from the clypeus. | 1A-B |
| far | metafurcal arm  [0000593] | The apodeme that arises from the metafurcal base of the metapectus, extends towards the metapleural apodeme and serves as the site of origin of metacoxal and metatrochanteral muscles. | 8E, 9A-B |
| fcl | lower face  [0000502] | The area that is limited dorsally by the antennal sockets, laterally by the compound eye and the malar groove and ventrally by the epistomal sulcus. | 1A-B, 1D, 2A |
| fcu | upper face  [0001044] | The area that is limited dorsally by the median ocellus, ventrally by the antennal sockets and medially by the inner margins of the compound eye. | 1A-B, 1D, 2A |
| fem | femur  [0000327] | The leg segment that is distal to the trochanter and proximal to the tibia. | 12A-D |
| fla | flagellomere  [0000342] | An article (separately, although passively, movable part) of the antenna, located distally of the pedicel on the multiannulated flagellum. | 2A, 3A-B |
| frs | frons  [0001523] | The entire region from the epistomal sulcus and up to the median ocellus, delimited laterally by the compound eye and the malar grooves. | 1A-B |
| fua | mesofurcal arm  [0000475] | The apodeme that arises from the base of the mesofurca. | 9A-B, 10A |
| fub | furcal base | The stem-like base of the apodeme (furca) that arises medially from the furcal pit on the ventral part of the thorax, fused anteriorly with the discrimenal lamella and dorsally to the furcal arms. | 9A-B, 10A, 10D |
| gaf | *gastral fold** | A more or less distinct lateral longitudinal crease, ridge or fold on abdomen, defining the border between the mediotergite and laterotergite. | 14A, 15A |
| gal | galea  [0000368] | The lobe that is located on the maxilla distal to the stipes and lateral to the lacinia. | 1A, 2B |
| gen | gena  [0000371] | The area that is delimited by the intersection of the interorbital plane, the margin of the compound eye, the margin of the oral foramen and, when present, the occipital carina and the malar sulcus. | 1A, 2A-B |
| glo | glossa  [0000376] | The median lobe that is unpaired and situated on the labium distally of the salivarial orifice. The glossa is the median labial endite. | 1A, 2B, 4A |
| gly | glymma | A lateral, longitudinal impression ventral to the lateral petiolar carina, anteriorly occasionally equipped with a laterope. | 13B |
| gon | gonoforceps  [0000381] | The sclerite that is the apical-most sclerite of the external male genitalia and that is connected along its basal margin with the cupula. | 15B, 16B, 17B, 17D |
| gsp | gastral spiracles | The spiracles that are located on the metasoma, posterior to the petiole. | 14A, 15A-B |
| gul | gula | The area that is located between the oral and occipital foramina, that is delimited laterally by the gular sulci or gular ridges when present, and that usually carries different microsculpture than the surrounding areas of the cranium. | 2C |
| ham | hamulus  [0000394] | A seta that is located on the anterior margin of the hind wing and interlocks with the recurved posterior edge of the fore wing in Hymenoptera during flight, making them functionally one-winged | 11B |
| hca | hypostomal carina  [0000413] | The carina that delimits the hypostoma from the cranium. | 2B-C |
| hpd | hypandrium | The last abdominal sternum of male wasps, forming the subgenital plate. | 15A, 15B |
| hst | hypostoma  [0000411] | The area that extends on the posterior (ventral) margin of the oral foramen along the site of attachments of the conjunctiva connecting the cranium with the maxilla, and that is delimited dorsally by the hypostomal carina and laterally by the pleurostomal acetabulum. | 2B-C |
| huc | humeral complex | The anatomical cluster that is composed of median plates in the wing base; a complex of sclerites that originates from different fore- and hind wing vein bases. | 11C-D |
| hum | *Hypocnemium** | The area the constitutes the lower part of the epicnemium | 5A, 7A |
| hyp | hypopygium | The last abdominal sternum of female wasps, forming the subgenital plate. | 14A |
| hyt | hypostomal tooth  [0000416] | The projection that is located on the posterolateral margin of the oral foramen at the junction of the hypostoma and the pleurostomal acetabulum. | 2E |
| inv | invagination for the occlusor muscle apodeme | The pit that corresponds with the occlusor muscle apodeme of the mesothoracic spiracle. | 5A |
| jug | jugal lobe | The posteroproximal lobe of the fore wing, behind the first anal vein 1A. | 11C |
| laa | lateral axillar area  [0000468] | The axillar area lateral (distal) to the axillar carina. | 5A, 6A, 10B-C |
| lab | labrum  [0000456] | The preoral, unpaired, dorsomedian lobe of the mouthparts that often is concealed under or partly to entirely exposed below the clypeus; suspended from the clypeus with muscles arising to the cranium. | 1A |
| lap | *lateropal apophysis** | The apophysis marked externally by the laterope. | 13C |
| lat | laterope | A pit at the anterior end of the glymma. | 13B |
| lft | lateral flag of the anterior tentorial arm | A membranous lateral extension of the anterior tentorial arm. | 1C |
| lml | lateral mesoscutal lobe  [0000466] | The area that is located between the notaulus and the parascutal carina. | 5A, 6A |
| lmp | lateral metepisternal projection  [0001403] | The projection that is situated anterolatelly on the metasubpleuron. | 5A, 7A, 8A, 8E |
| lpa | labial palp  [0000450] | The palp that is situated on the labium, articulating basally with the lateral part of the prementum. | 1A, 2A-B, 4A |
| lpc | lateral petiolar carina | A carina running longitudinally from the petiolar collar along the lateral margin of the petiole back to the petiolar spiracle. It marks the dorsal margin of the glymma and laterope, when present. | 13B, 13E-F |
| lpp | lateral propleural area  [0000484] | The dorsally inflected area located laterally on the propleuron, forming an obliquely vertical surface. | 8C |
| mac | mandibular acetabulum  [0001391] | The acetabulum that is located dorsolaterally on the proximal edge of the mandible, and that accommodates the pleurostomal condyle. | 4B-C |
| mae | mesopostnotal apodeme | The anterolateral apodeme extending from the mesopostnotum towards the posterodorsal corner of the mesopectus. | 10C, 10E |
| man | manubrium  [0000671] | The sclerite that is located proximodorsally on the pretarsus and connects the distodorsal margin of the telotarsus with the dorsal part of the arolium. | 12H |
| map | mesopleural apodeme  [0001359] | The apodeme that serves as the site of origin of the second mesopleuro-mesonotal and the mesopleuro-mesocoxal muscles. | 10A, 10D |
| mba | metabasalar apodeme | A protruding anterolateral apodeme on the venterolateral edge of the paracoxal ridge, serves as the site of origin of the anterolateral metapleuro-metabasalar muscle. | 8E |
| mcc | mesocoxal condyle  [0001389] | The distolateral process of the internal rim of the mesocoxal foramen, which articulates with the acetabulum of the mesocoxa. | 7A, 8A |
| mcf | mesocoxal foramen  [0001785] | The coxal foramen that is located on the mesopectus. | 7A, 8A, 9B |
| mco | mandibular condyle  [0000508] | The condyle that is located venterolaterally on the proximal edge of the mandible and inserts into the pleurostomal acetabulum. | 4B-D |
| mcp | *mesocoxal process** | The lateral process of the mesocoxa articulating distally with the coxal condyle of the mesopectus (the mesocoxal condyle). | 12D |
| mdi | mesodiscrimen  [0000545] | The longitudinal sulcus on the ventral surface of the mesothorax that corresponds to the mesodiscrimenal lamella. | 7A, 8A, 10A |
| mdl | mesodiscrimenal lamella  [0000546] | The discrimenal lamella that is located in the mesothorax. | 9B, 10A, 10D |
| med | metascutellar disc  [0000625] | The area that is located posteromedially on the metanotum, is delimited laterally by the metascutellar trough and corresponds to the reservoir of the dorsal vessel. | 5A, 6A, 8A |
| mef | *mesepimeral flange**  [0000751] | The posterior area of the mesepimeron, behind the mesepimeral sulcus. | 5A |
| mer | mesepimeral ridge  [0000537] | The ridge that extends along the posterior margin of the mesopectus. | 9A-B, 10A, 10D |
| mes | mesepisternum  [0001872] | The anterior subdivision of the mesopleuron, anterior to the mesopleural sulcus. | 5A, 6A, 7A |
| met | *metascutellar trough**  [0000600] | The depressed area lateral to the metascutellar disc. | 6A |
| mfb | mesofurcal bridge  [0000548] | The apodeme that connects the lateral mesofurcal arms and serves as the site of origin of the dorsal mesofurco-profurcal muscle. | 9A-B |
| mfp | mesofurcal pit  [0000549] | The furcal pit that is located on the ventral surface of the mesothorax. | 7A, 8A, 10A |
| mfu | mesofurca  [0000547] | The furca that arises from the mesopectus and is continuous with the mesodiscrimenal lamella. | 10A |
| mgr | malar groove  [0001394] | The groove that extends between the compound eye and the base of the pleurostoma. | 1A, 2A |
| mla | *mandibular lancea** | The spear-like ventral flange of the mandible, issuing from the mandibular condyle and ending apically in a more or less sharp point. | 4B-D |
| mlo | *mesepimeral lobe** | The area that is located posterodorsally on the posterior mesepimeral area and obscures the metathoracic spiracle. | 5A |
| mmd | medio-posterior mesoscutal depression  [0000637] | The pit- or groove-like depression where the notauli join posteriorly. | 6B |
| mml | median mesoscutal lobe  [0000520] | The median anteromesoscutal area that is located between the notauli. | 5A, 6A |
| mmt | mesal membrane of the anterior tentorial arm | The U-shaped membrane that is attached anteroventrally along and between the two anterior tentorial arms. | 1C |
| mna | mesoscutellar apodeme | The large, tubercle-shaped apodeme corresponding to the mesoscutellar pit at the distal end of the mesoscutellar trough. | 10A-C, 10E |
| mnd | mandible  [0000506] | The sclerite that is connected to the cranium along the anterior margin of the oral foramen via the anterior and posterior cranio-mandibular articulations. | 1A-B, 2A, 4B-F |
| mnn | median metanotal notch | The notch that is median and is located on the posterior margin of the metanotum. | 6A |
| mno | mesonotum  [0000506] | The area that is limited anteriorly by the pronotum, laterally by the basalare, axillary sclerites and subalare and posterolaterally by the mesopostnotum and the metanotum. | 5A, 6A |
| mnp | metanotal pit | The deep, apophysis-marking pit situated anterolaterally of the metascutellar trough. | 6A |
| mpa | maxillary palp  [0000515] | The palp that is located on the maxilla articulating basally with the lateral part of the stipes. | 1A, 2A-B, 4A |
| mpb | mesopleural scrobe  [0001358] | The horizontal sulcus that is situated posteriorly on the lateral surface of the mesopectus, and that corresponds internally to the mesopleural apodeme, which is the site of origin of the mesopleuro-mesocoxal and the second mesopleural-metanotal muscles. The mesopleural scrobe is likely to be a short remnant of the primitive mesopleural sulcus defining the boundary between the mesepisternum and the mesepimeron. | 5A, 8A |
| mpc | mesopectus  [0000557] | The huge mesosomal sclerite that is U-shaped in cross section; that originates from the merged mesopleura and mesosternum; that is connected anteriorly with the pronotum and the propectus, dorsally with the basalare, the mesonotum, the second axillary sclerite and the subalare, and posteriorly with the metapectus; and that houses the mesodiscrimenal lamella and the mesofurca. | 5A, 7A, 8A, 9B, 10A |
| mpe | metapectus | The ventral part of the metapectal-propodeal complex, which originates from the merged metapleura and metasternum. The suture separating the metapectus and the propodeum is most commonly rather faint, but if visible it runs anteroventrally to the propodeal spiracle. | 5A, 6A, 7A, 8A, 9B |
| mph | mesophragma  [0000558] | The phragma that extends along the ventral (posterior) margin of the ventral mesopostnotal flange and serves as the site of origin of the prophragmo-mesophragmal and the mesophragmo-metaphragmal muscles. | 10A-C, 10E |
| mpi | *mesoscutellar pit** | The deep, apophysis-marking pit situated anterolaterally of the mesoscutellar trough, just mesal to the sclerite’s keel-like postalar process. | 6A |
| mpn | mesopostnotum  [0000567] | The sclerite that bears the mesophragma and the mesolaterophragma. | 10B-C, 10E |
| mpp | multiporous plates  [0001731] | A patch that is elevated, located on the flagellomere and is multiporous. | 3F-G |
| mpr | *metapectal-propodeal rim** | The reinforced rim that surrounds the propodeal and metacoxal foramina. | 7A, 8A |
| mps | marginal pronotal sulcus | The sulcus which runs along the outer margin of the pronotum. | 5A, 7A |
| msc | mesoscutum  [0000575] | The part of the mesonotum anterior the scutoscutellar suture, i.e. the anteromesoscutum and the axilla combined. | 5A, 6A |
| msd | mesoscutellar disc  [0000915] | The dorsal area of the mesoscutellum that is delimited anteriorly by the scutoscutellar sulcus and laterally by the mesoscutellar trough and that internally houses the pulsatory organ of the fore wing. | 5A, 6A, 8A, 10A |
| msp | malar space  [0000503] | The shortest distance between the compound eye and the ventrolateral margin of the cranium. | 1A, 2A |
| msu | mesepimeral sulcus  [0000538] | The sulcus that extends along the posterior margin of the mesopectus, delimits the mesepimeral area and corresponds to the mesepimeral ridge. | 5A |
| mta | metapleural apodeme | The apodeme that corresponds externally to the metapleural scrobe. | 10A |
| mtb | *metapleural scrobe** | The horizontal anterolateral sulcus on the metapectal-propodeal complex. It may be a part or remnant of the primitive metapleural sulcus. | 5A |
| mtc | metacoxal condyle  [0001385] | The distolateral process of the internal rim of the metacoxal foramen, which articulate with the acetabulum of the metacoxa. | 7A, 8A |
| mte | mandibular teeth  [0001019] | The projections that are located distally on the mandible. | 4B-4D |
| mpf | metanotal-propodeal fissure | The transverse fissure that extends between the metanotum and the metapectal-propodeal complex. | 5A |
| mtn | metanotum  [0000603] | The tergum of the metathorax, in Hymenoptera situated between the mesoscutellum and the metapectal-propodeal complex. | 5A, 6A, 8A, 10A |
| mtp | metafurcal pit  [0000594] | The furcal pit that is located on the ventral surface of the metathorax or the metapectal-propodeal complex. | 7A, 8A |
| mts | metapleural sulcus | The line that corresponds to the metapleural ridge. | 5A, 6A, 8A |
| mtw | metanotal wing process  [0001326] | The acetabulum that is located anteroventrally on the posterior supraalar area and accommodates the posterior condyle of the first axillary sclerite of the hind wing. | 5A, 6A |
| mum | mesoscutellum  [0000574] | The part of the mesonotum posterior to the scutoscutellar sulcus. | 5A, 6A |
| mxf | metacoxal foramen  [0001264] | The coxal foramen that is located on the metapectus. | 7A, 8A, 9B |
| mxp | *metacoxal process** | The lateral process of the metacoxa articulating distally with the coxal condyle of the metapectal-propodeal complex (the metacoxal condyle). | 12C |
| not | notauli  [0000647] | The pair of posteriorly converging lines on the mesoscutum, which correspond to the median border of the site of origin of the first mesopleuro-mesonotal muscle and divides the mesoscutum into one medial and two lateral lobes. Sometimes incorrectly termed "parapsidal furrows". | 5A, 6A, 10A |
| oca | occipital carina  [0000643] | The carina that dorsolaterally surrounds the occiput. | 2A-B |
| occ | occiput  [0000643] | The area that is located posteriorly on the cranium; that extends anteriorly to the posterior margin of the vertex and the gena, the border sometimes being marked by an occipital carina; and that medially encircles the postocciput. | 2B |
| ocf | occipital foramen  [0000643] | The foramen that is located posteriorly on the head and is delimited dorsally by the postocciput. | 2B-C |
| ocl | ocellus  [0000661] | A simple eye that is located on the top of the head, composed of the corneal lens, pigment cell, rhabdoms and synaptic plexus. | 1A-B, 1D, 2A-B |
| oco | occipital condyle  [0000654] | The condyle that is situated on the postocciput and that articulates with the cervical prominence. | 2C |
| oma | occlusor muscle apodeme of anterior thoracic spiracle  [0000659] | The apodeme that serves as the site of origin of anterior thoracic spiracle occlusor muscle. | 9A, 10A |
| osu | ocular sulcus  [0000664] | The sulcus that extends along the margin of compound eye and corresponds to the circumocular ridge. | 1A, 1D, 2A |
| ovs | ovipositor sheath  [0001012] | The sclerite that is located posterior to the second valvifer, is connected to the second valvifer via conjunctiva, and protects the first and second valvulae when they are not used. | 14A, 16A, 17A, 17C |
| pac | propodeal acetabulum | The acetabulum that is located on the mesal edge of the metapectal-propodeal rim, beneath the propodeal tooth, and articulates with the petiolar condyle. | 8A |
| pap | postalar process  [0000742] | The process that is located anterolaterally on the mesoscutellar-axillar complex. | 5A, 6A |
| par | parossiculus  [0000703] | The sclerite that is connected distally with the gonossiculus, laterally with the gonostipes, and proximally with the gonostipital arm. | 15B, 16B, 17B, 17D |
| pax | preaxilla  [0000800] | The area that is set off from the anteromesoscutum by the parascutal carina, that is adjacent to the tegula anteriorly, and that bears the anterior and antemedian mesonotal wing processes. | 5A |
| pba | *posterior bar of metascutellum*  [0000623] | The raised bar, running along the posterior margin of the metanotum, posterior to the metascutellar trough. | 6A |
| pbm | posterior bar of mesoscutellum  [0001903] | The raised and hollowed bar, running along the posterior margin of the mesoscutellum, posterior to the mesoscutellar trough. | 6A |
| pbs | probasisternum  [0001317] | The horizontal area of the prosternum anterior to the transverse sternacostal carina or sternacostal suture, and the site of origin of the prodiscrimen. | 7A, 8B |
| pcf | procoxal foramen  [0001315] | The coxal foramen that is located on the propectus. | 7A |
| pco | petiolar condyle | The anterolateral condyle of the petiole, which articulates with the propodeal acetabulum. | 13A-C, 13E-G, 14A, 15A |
| pcp | *procoxal process** | The lateral process of the procoxa articulating distally with the coxal condyle of the propleuron. | 12A |
| pcr | paracoxal ridge  [0000684] | The ridge that extends along the anterior margin of the metapectus. | 8E, 9A |
| pcs | paracoxal sulcus  [0000685] | The sulcus that extends along the anterior margin of the metapectus and corresponds to the paracoxal ridge. | 7A |
| pdi | prodiscrimen  [0000823] | The longitudinal sulcus that is situated on the ventral surface of the propectus and corresponds to the prodiscrimenal lamella. | 7A, 8B |
| pec | *petiolar collar**  [0001734] | The obliquely dorsally and laterally projecting anterior flange of the petiolar tergum, which bears the petiolar acetabula. | 13A, 13F |
| ped | pedicel  [0000706] | The antennal segment that is the second segment of the antenna and is connected proximally with the scape and distally with the flagellum. | 2A, 3A-B |
| pef | *petiolar fovea**  [0000129] | A lateral depression on the dorsal surface of the petiolar collar. | 13A |
| pen | penisvalva  [0000707] | The sclerite that is paired, located in the middle of the external male genitalia, and surrounds the distal part of the ductus ejaculatorius and the endophallus. | 15B, 16B, 17B, 17D |
| pes | petiolar spiracle  [0001538] | The spiracle that is located on abdominal segment 2. | 13A-B, 13E-F, 14A, 15A |
| pet | petiole  [0000020] | In Hymenoptera: Apocrita the second abdominal (first metasomal) segment, often stem- or stalk-like, anteriorly attached to the propodeum and posteriorly to the third abdominal segment, which may also be stem- or stalk-like (some Formicidae). | 13A-F, 14A, 15A |
| pfa | profurcal arm  [0000826] | The sternal apophysis that is paired, arises from the furcasternum of the prothorax and is continuous with the prodiscrimenal lamella. | 8B-C |
| pfb | profurcal base | The basal stem-like part of the profurca. | 8B-C |
| pfo | propodeal foramen  [0000865] | The foramen that is located on the metapectal-propodeal complex, and into which the anterior end of the petiole is inserted. | 8A |
| pfs | profurcasternum  [0001310] | The vertical area of the prosternum posterior to the transverse sternacostal carina or sternacostal suture, and the site of origin of the profurcal base. | 7A, 8B-C |
| pge | postgena  [0000776] | The area that is located on the occiput ventrally of the occipital foramen. | 2B |
| pgl | paraglossa  [0000686] | The lobe that is submedian, paired, and is situated distally on the labium, laterally of the glossa. | 2B |
| pha | phallus  [0000312] | The anatomical cluster that is composed of the cupula, gonostyle, volsella and the aedeagus. | 15B |
| pla | planta  [0000719] | The sclerite that is located proximoventrally on the pretarsus and is connected proximally with the unguitractor plate and distally with the arolium. | 12G |
| plc | pleurostomal carina  [0001293] | The carina that delimits the pleurostoma from the cranium. | 1A, 2A |
| plo | pronotal lobe  [0000836] | The area that is located dorsally on the posterior margin of the pronotum and projects over the first thoracic spiracle. | 5A, 6A, 9B |
| pls | pleurostoma  [0000730] | The cranial area that is delimited by the clypeus, gena and hypostoma, and that is equipped with the two articular points of the mandibles: the pleurostomal acetabulum and pleurostomal condyle. | 1E, 2B, 2D |
| pma | propleural marginal area | The anteroventrally edge of the propleuron, posteriorly delimited by a delicate propleural cervical sulcus. | 7A, 8B |
| pno | pronotum  [0000853] | The notum that is located in the prothorax. | 5A, 6A, 7A, 9A-B, 10A |
| pnp | pronope  [0000830] | The depression situated mediodorsally on the pronotum. | 6C, 9B |
| poc | postocciput  [0000790] | The area that is delimited by the postoccipital sulcus or postoccipital carina and the occipital foramen, and that is equipped with the occipital condyles. | 2B-C, 2E |
| pom | postmentum  [0000785] | The sclerite that is located proximally to the prementum, and that serves as origin of the postmento-premental muscle. | 2B, 4A |
| pos | postoccipital suture  [0000789] | The line that delimits the postocciput from the cranium. | 2C |
| ppa | posterior propleural arm  [0000856] | The projection that is situated dorsally on the posterior part of the lateral propleural area, and extends posteromesally. | 8B-C |
| ppf | propleural flange  [0000295] | The often flap-like area that is located posteroventrally on the propleuron. It may be delimited anteriorly by an epicoxal sulcus and it may be curved up over the ventral apex of the pronotum. | 5A, 7A, 8B-C |
| pph | pseudophragma  [0000881] | The flange that extends along the dorsal margin of the mesopostnotum. [1187] | 10B-C, 10E |
| ppi | posterior pronotal inflection  [0000761] | The pronotal inflection that extends along the posterodorsal and lateral pronotal margin, that serves as an interior locking mechanism between the pronotum and the anterior margin of the mesopectus, and that is externally marked by the posterior section of the marginal pronotal sulcus. | 9A 10A |
| ppl | propleuron  [0000862] | Pleuron of the prothorax. | 5A, 7A, 8B |
| ppo | propodeum | The first abdominal segment in Hymenoptera: Apocrita, where it is merged with the metapectus to form the last sclerite of the functional thorax (the alitrunk or mesosoma). | 5A, 6A, 8A, 10A |
| ppr | prespecular ridge | The ridge that delimits the site of origin of the anterior mesopleuro-mesofurcal muscle | 9B |
| pps | propodeal spiracle  [0000329] | The spiracle of the propodeum. | 5A, 6A, 8A |
| ppt | propodeal tooth  [0000866] | The tooth that is located on the mesal edge of the metapectal-propodeal rim, above the propodeal acetabulum. | 8A |
| pre | prementum  [0000804] | The sclerite that is unpaired and is situated ventrally on the labium proximal to the apical lobes (the labial endites) and the palps. The sclerite laterally extends to the hypopharyngeal wall. | 2B, 4A |
| pri | pronotal inflection | The strengthened rim of the anteroventral edge of the pronotum, i.e. the margin facing the head and the propleura. Externally marked by the anterior and anteroventral section of the marginal pronotal sulcus. | 9A, 10A |
| prp | propectus  [0000854] | The anatomical cluster that consists of the propleura and prosternum. | 7A |
| prs | prespecular sulcus | The sulcus that delimits anteriorly the speculum and corresponds to the anterior margin of the speculum. | 5B |
| prt | pretarsus  [0000820] | The anatomical cluster that is apical to the telotarsus and composed of the empodium, auxilia, planta, pulvillum, unguis, unguitractor plate, auxiliar sclerite and manubrium. | 12A-C |
| psf | parascutal flange  [0000697] | The submarginal rim that extends along the mesonotal lateral margin, separated from the remainder of the mesoscutum by a parascutal sulcus. | 5A, 6A |
| psi | parastigma | A sclerotized area at the anterior fore wing margin, basal to the pterostigma, often consisting simply of an extension of the first abscissa of the radial sector of the fore wing (1RS). | 11A |
| pss | parascutal sulcus  [0000569] | The sulcus that extends medially along the mesonotal lateral margin, separating the parascutal flange from the remainder of the mesoscutum. | 5A, 6A |
| pst | prosternum  [0000873] | The posterodorsal sclerite of the propectus, divided by the transverse sternacostal carina or sternacostal suture into an anterior, horizontal basisternum and a posterior, vertical profurcasternum (or sternellum). | 7A, 8B |
| pta | posterior tentorial arm  [0001343] | The apodeme that extends between the site of origin of the tentorial bridge and the posterior tentorial pit. | 1C |
| ptp | posterior tentorial pit  [0000768] | The tentorial pit that corresponds to the posterior tentorial arm. | 1C, 2B-C, 2E |
| rad | radicle  [0000889] | The area that is situated proximally on the scape, that is limited distally by a constriction and that is equipped proximally with the antennal articular process. | 2A, 3A-B |
| S(X) | abdominal sternum (number)  [0001425] | A sternum of an abdominal segment. | 14A, 15A-B, 16B |
| S2a | anterior area of the second abdominal sternite | In Ichneumonoidea: the anterior, well sclerotized area of the second abdominal sternum. | 7A, 13H, 14A, 15A |
| S2b | posterior area of the second abdominal sternum | In Ichneumonoidea: the posterior, membranous area of the second abdominal sternum. | 14A, 15A |
| saa | subalar area  [0000960] | The area that is situated dorsally on the mesepimeral flange, that is often shaped like a strengthened rim, and that bears the subalar tubercle. | 5A, 6A |
| sac | scutellar-axillar complex  [0000572] | The mesonotal area that is located posteriorly of the transscutal articulation and is composed of the axillae and the mesoscutellum. | 5A, 6A |
| sai | subalar impression | The depression that is situated anteriorly between the subalar area and the subalar bridge. It becomes deeper and deeper posteriorly and ends as a deep pit at the junction between the subalar area and the subalar bridge. | 5A |
| sat | subalar tubercle  [0001475] | The projection that is located posteriorly on the subalar area and that accommodates the posterior end of the second axillary sclerite of the fore wing at the beginning of the upstroke of the wing. | 5A |
| scc | *sternacostal carina** | The transverse carina on the prosternum that separates the horizontal probasisternum from the vertical profurcasternum. | 7A, 8B-C |
| scp | scape  [0000908] | The first, basal antennal segment that proximally articulates with the cranium and distally with the pedicel. | 2A, 3A, 3B |
| sct | *mesoscutellar trough**  [0001161] | The lateral, depressed area between the mesoscutellar disc and the mesoscutellar arm, ending anterolaterally in the mesoscutellar pit. | 5A, 6A |
| spe | speculum  [0000944] | The area that is located dorsally on the mesopleuron, is delimited posteriorly by the mesepimeral ridge and posteroventrally by the mesopleural scrobe and corresponds to the site of origin of the dorsal mesopleuro-mesofurcal muscle. | 5A |
| spl | metasubpleuron | The ventral region of the metapectus | 7A, 8A, 8E, 9A-B, 10A |
| ssr | scutoscutellar ridge  [0000918] | The ridge that extends along the anterior margin of the mesoscutellar disc and corresponds to the scutoscutellar sulcus. | 10B-C, 10E |
| sss | scutoscutellar sulcus  [0000919] | The sulcus that corresponds to the scutoscutellar ridge. | 5A, 6A |
| stg | (ptero)stigma | A sclerotized, often pigmented area extending from the anterior margin of the fore wing, distal to the apex of the costal vein | 11A |
| sti | stipes  [0000958] | The sclerite that is located in the maxilla and articulates proximally with the cardo, distally with the galea and lacinia, and laterally with the maxillary palp. | 2B, 4A |
| str | strigil  [0000102] | The anatomical cluster that consists of the probasitarsal comb and calcar. | 12A, 12E |
| sub | *subalar bridge** | A crest-like reinforcement beneath the subalar impression, connecting the mesepimeral flange to the mesepisternum. | 5A |
| sup | subalar pit  [0000961] | The pit that is located posterodorsally on the mesopectus, ventrally to the mesepimeral flange, and that corresponds to a large internal apophysis at the dorsal end of the mesepimeral ridge. | 5A |
| T(X) | abdominal tergum (number)  [0001426] | A tergum that is located in the abdomen. | 14A, 15A, 16A-B |
| T2a | mediotergite of petiole | In Ichneumonoidea: the dorsal area of the second abdominal tergum, separated on each side by the gastral fold from the corresponding laterotergite. | 13A-G, 14A, 15A |
| T2b | laterotergite of petiole | In Ichneumonoidea: the lateral area of the second abdominal tergum, separated from the mediotergite by the gastral fold. | 13G, 14A, 15A |
| tar | tarsus  [0000992] | The leg segment that is apical to the tibia. | 12A-C |
| tax | *triangular axillar region** | The area that is the lateral surface of the dorsal axillar area, defined laterally by the axillar carina and mesally by a proposed line formed by anterior prolongation of the lateral margin of the mesoscutellar disc. | 6A |
| tbr | tentorial bridge  [0000998] | The apodeme that medially connects the tentorial arms. | 1C |
| teg | tegula  [0000993] | The sclerite that is located laterally of the preaxilla and obscures the anterior mesonoto-first axillary and the mesopleuro-second axillary sclerite articulations. | 5A, 6A, 9A |
| ten | tentorium  [0001003] | The apodeme that has its sites of origin marked by the anterior and posterior tentorial pits and the gular sulci. | 1B, 1C |
| ter | terebra  [0001004] | The anatomical cluster of female genitalia that is composed of the first and second valvulae. | 14A, 16A, 17A, 17C |
| tib | tibia | The leg segment that is proximal to the tarsus and distal to the femur. | 12A-C, 12E-F |
| tps | transverse pronotal sulcus  [0001032] | The transverse sulcus of the pronotum that corresponds to the anteromedian pronotal ridge. | 5A |
| tro | trochanter | The leg segment that is located proximal to the femur and distal to the [coxa](http://api.hymao.org/projects/32/public/ontology_class/show/559). | 12A-D |
| tru | trochantellus | The area that is located proximally on the femur. It is distally delimited, at least partly, by an impression or sulcus and articulates basally with the trochanter. | 12A-D |
| tsa | transscutal articulation  [0001204] | The transverse line of separation that divides the mesonotum into an anterior anteromesoscutum and a posterior scutellar-axillar complex. | 5A, 6A |
| tsp | tibial spur  [0001018] | A spur that is located distally on a tibia. | 12B-C, 12F |
| tta | telotarsus  [0000994] | The tarsomere that is apical | 12A-C, 12G-H |
| unf | unguifer  [0001042] | The area that is located distomedially on the end of the telotarsus and serves as the site of articulation for the pretarsal claws. | 12G-H |
| ung | unguitractor  [0001043] | The sclerite that serves as the site of insertion of the flexor of the pretarsus. | 12G |
| val1 | first valvula  [0000339] | Gonapophysis of the eighth abdominal segment, which forms the ventral portion of the terebra (or ovipositor stylets); the sclerite that is distal to the first valvifer and articulates with the second valvula via the aulax. | 17A |
| val2 | second valvula  [0000928] | Gonapophysis of the ninth abdominal segment, which forms the dorsal part of the terebra (or ovipositor stylets); the area that is continuous proximally with the second valvifer via the ventral ramus of the second valvula, articulates with the second valvifer via the basal articulation and with the first valvula via the olistheters. | 17A |
| vlf1 | first valvifer  [0000338] | A small sclerite connected to the base of first valvula and the abdominal tergum 9 | 17A, 17C |
| vlf2 | second valvifer  [0000927] | A small sclerite connected proximally to the second valvula | 17A, 17C |
| vol | volsella  [0001084] | The anatomical cluster that is composed of the parossiculus and digitus. | 15B, 16B, 17B, 17D |
| vtx | vertex  [0001077] | The dorsal area of the head, between the inner margins of the compound eyes. It is delimited anteriorly by the anterior ocellus and posteriorly by the occiput. | 1D, 2A-B |
| vvu | valvura  [0001050] | The area that is located proximally of the ergot on the penisvalva | 16B, 17B, 17D |
